# *One-Pot* Solvent-Involved Synthesis of 5-*O*-Substituted 5*H*-Chromeno[2,3-*b*]pyridines

**DOI:** 10.3390/molecules28010064

**Published:** 2022-12-21

**Authors:** Yuliya E. Ryzhkova, Fedor V. Ryzhkov, Michail N. Elinson, Oleg I. Maslov, Artem N. Fakhrutdinov

**Affiliations:** N. D. Zelinsky Institute of Organic Chemistry Russian Academy of Sciences, 47 Leninsky Prospekt, 119991 Moscow, Russia

**Keywords:** *one-pot* reaction, chromeno[2,3-*b*]pyridine, salicylaldehyde, alcohol, malononitrile dimer

## Abstract

Chromeno[2,3-*b*]pyridines are substances demanded in medicinal and material chemistry. *PASE* (pot, atom, and step economy) and in particular *one-pot* approaches are key green chemistry techniques that are applied for the synthesis of heterocyclic compounds. In this case, the *PASE* approach was extended with ‘component economy’, as solvent was used also as reactant (solvent-involved reaction). This approach was adopted for the *one-pot* synthesis of previously unknown *O*-substituted 5-alkoxy-5*H*-chromeno[2,3-*b*]pyridines via *two-step* transformation, namely the reaction of salicylaldehydes and malononitrile dimer, with the subsequent addition of alcohol. The mechanistic studies revealed the possibility of concurrent reaction. The studies aided in optimizing the reaction conditions for the best yields (77–93%). Thus, the *one-pot* reaction proceeds efficient and quickly, and the work-up procedure (only simple filtering) is very convenient. The structure of synthesized chromeno[2,3-*b*]pyridines was confirmed by 2D NMR spectroscopy.

## 1. Introduction

Global ecological awareness has made green chemistry a broad, beneficial and promising area of organic chemistry [1,2]. Green chemistry is based on 12 principles [1] that lead to the development of the simplest synthetic approach with the utilization of the least number of components (the less toxic, the better); the result should carry the least number of by-products and environmental threats.

The current level of chemistry does not allow all 12 principles to be applied to resolve all drawbacks and limitations (for example, avoiding solvents or special precursors) in the development of every new process. Thereby, in synthetic chemistry, the *PASE* approach has become widespread [3,4,5,6,7] and it is currently a key green technique [4]. This approach is focused on the economy (E) of pot (P), atom (A) and steps (S). Atomic economy states that the majority of atoms in a reaction become a component of the final product. This concept prevents waste; however, if the waste is inevitable, according to green chemistry, water is the best by-product. Step economy is linked to the energy efficiency principle; it is preferable to have the fewest stages (separate transformations, work-ups) [8] and to have all transformations in one pot [8,9].

Another green principle is to utilize a less toxic solvent or completely reduce it [10]. Solvent-free reactions [11,12], solid-state reactions [13,14,15]—in case of solid aldehydes, on-water (water-assisted), and reactions in emulsions [12,16,17,18] are examples of green methods with varying solvent roles that were developed with that principle. These approaches greatly reduce the amount of solvent, increase the reaction rate [19,20] and produce excellent yields [15,16,20].

Among the approaches mentioned above, reactions in which solvents are involved in the transformations [21] have never received special attention. However, it would be a consistent extension of such approaches. More than that, the utilization of solvent as a reactant is applied in polymer [22] and organic syntheses (Friedel–Crafts acylation) as they possess several advantages: they are simple, reduce waste, and are easy to work up.

Chromenopyridine derivatives have a wide spectrum of properties that are useful for medicinal chemistry. Among them, glucocorticoid receptor activity [23], antiproliferative [24], anti-tumor [25] anti-rheumatic [26], anti-histaminic [26] and anti-asthmatic [27] properties are shown. Pranoprofen is a nonsteroidal anti-inflammatory drug with analgesic and antipyretic actions [28].

**Figure 1:**
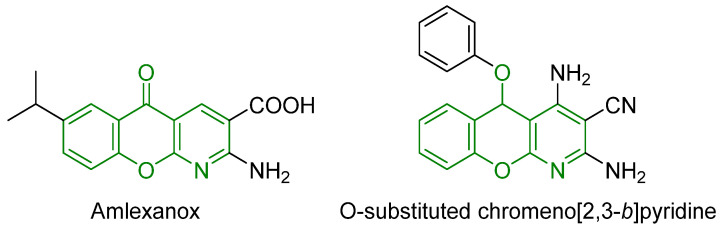
5-*O*-chromeno[2,3-*b*]pyridines with proven useful properties.

Chromenopyridine derivatives with an additional atom of oxygen at the 5-position are a more specific class of compounds. Amlexanox (Figure 1) is an anti-allergic drug, clinically effective for atopic diseases, especially allergic asthma and rhinitis [29]. Another practically useful compound of this class is 5-phenoxy-5*H*-chromeno[2,3-*b*]pyridine (Figure 1) [30], which, when placed on the surface of low-carbon steel, prevents its corrosion, even in a 15% hydrochloric acid solution [30]. There are some other derivatives with similar properties [31,32].

Among these compounds, the structure of 5-phenoxy-5*H*-chromeno[2,3-*b*]pyridine (Figure 1) provides the best potential for modification of the *O*-substitution. Furthermore, 5-phenoxy-5*H*-chromeno[2,3-*b*]pyridine is the only structure known to have an additional oxygen substitution [30]. At the same time, phenol, which is used for the synthesis, is quite a toxic component. It would be better to replace phenol fragments with less toxic and more cheap alcohols. However, the synthesis of 5-phenoxy-5*H*-chromeno[2,3-*b*]pyridine is carried out in ethanol [30], and no 5-ethoxy-5*H*-chromeno[2,3-*b*]pyridine products were obtained in this procedure.

We have accomplished several syntheses of chromeno[2,3-*b*]pyridines [5,33] and some green transformations with different roles of solvent, among them [12] on-solvent [12], on-water [18], and solid-state ([15] procedures. In addition to these green methods, we would like to present the first synthesis of *O*-substituted 5-alkoxy-5*H*-chromeno[2,3-*b*]pyridines, in which solvent is used as a reactant (solvent-involved—[21]) and plays a role in the final nucleophilic addition, becoming a fragment of the final structure. It delivers atom economy, reduces the number of used components, and thereby makes the whole process greener.

## 2. Results and Discussion

### 2.1. Formation of 5-Alkoxy-5H-Chromeno[2,3-b]pyridine ***4***

Previously we synthesized various types of 5-*C*- and 5-*P*-chromeno[2,3-*b*]pyridines [5,33,34,35,36,37,38]. In general, the interaction of a carbonyl group with a CH acid and the subsequent addition of another CH acid are easily feasible. Modifying CH acid to another nucleophilic agent, on the other hand, is a more difficult task. We discovered this for the first time in the synthesis of 5-*P*-chromeno[2,3-*b*]pyridines [5]. To complete the synthesis, we had to use an aprotic solvent (CH_3_CN), which enhances the nucleophilic properties of reactants.

After *C*- and *P*-substituted products were synthesized, we concentrated on 5-*O*-substituted chromeno[2,3-*b*]pyridines. It turns out that only a few examples are known, and only a few of them have been synthesized with *one-pot* or multicomponent methods [39,40,41].

**Scheme 1:**
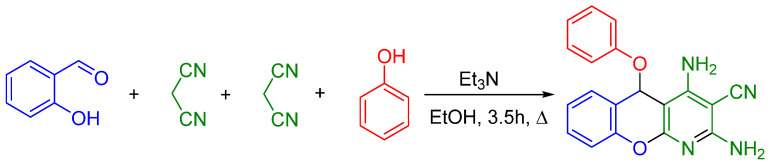
Multicomponent synthesis of 2,4-diamino-5-phenoxy-5*H*-chromeno[2,3-*b*]pyridine-3-carbonitrile.

One such example is the multicomponent synthesis of 2,4-diamino-5-phenoxy-5*H*-chromeno[2,3-*b*]pyridine-3-carbonitrile (Scheme 1) [30]. Among other things, it is difficult to assess the generality of the reaction, since the authors presented only one such compound. The mechanism of the reaction was also not described thoroughly.

**Scheme 2:**
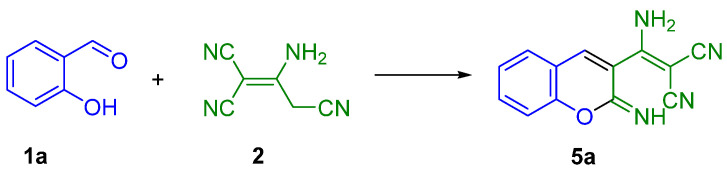
The transformation of salicylaldehyde **1a** and malononitrile dimer **2** into chromene **5a**.

We were encouraged to deeply investigate such processes in case of malononitrile dimer. First of all, we synthesized chromene **5a** for the mechanism investigation. However, during the recrystallization of chromene **5a** after the reaction (Scheme 2), some byproducts were found.

**Scheme 3:**
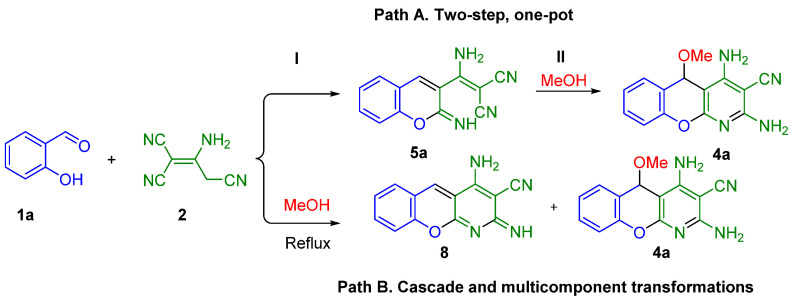
*Two-step one-pot* (or multicomponent*)* formation of 5-*O*-chromeno[2,3-*b*]pyridine **4a** and cascade formation of chromeno[2,3-*b*]pyridine derivative **8**. For the conditions see Table 1 in Section 2.3, Entry 1.

**Figure 2:**
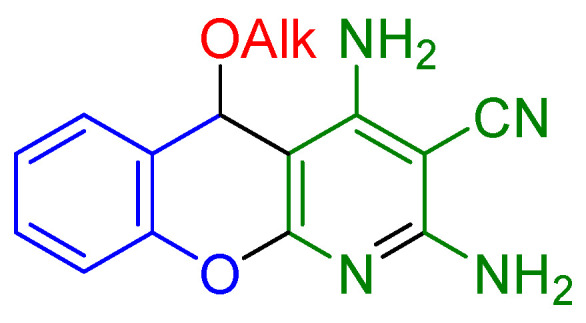
5-Alkoxy-5*H*-chromeno[2,3-*b*]pyridine **4**.

NMR analysis (see Section 2.4 for more detailed analysis) revealed that harsh recrystallization results in the formation of 5-alkoxy-5*H*-chromeno[2,3-*b*]pyridine (Figure 2, Scheme 3 in Section 2.2).

Thus, in this article, we present a mechanistic investigation of the formation of 5-alkoxy-5*H*-chromeno[2,3-*b*]pyridine with proven structure (2D NMR) as well as a convenient approach to the new substituted 5-*O*-chromeno[2,3-*b*]pyridines.

### 2.2. Mechanism of Formation of 5-Methoxy-5H-Chromeno[2,3-b]pyridine ***4a***

In this case, the addition of MeOH to chromene **5a** appears to allow the formation of 5-*O*-chromeno[2,3-*b*]pyridine. We have carried out several *one-pot* transformations to prove this (Scheme 3).

A *two-step* transformation results in 5-*O*-chromeno[2,3-*b*]pyridine **4a** after the complete formation of intermediate **5a** (it precipitates, then redissolves) (Scheme 3). The intermediate **5a** was detected in the reaction mixture (NMR). The steps of the *two-step* transformation were controlled by temperature. The final cyclization to **4a** (step **II,** Scheme 3) demands heating at reflux.

If the heating is provided immediately without complete formation (precipitation) of intermediate **5a,** the process affords a mixture of compounds **8** and **4a**. Thus, in this case 5-*O*-chromeno[2,3-*b*]pyridine **4a** is formed in a multicomponent reaction, but concurrent cascade formation of chromeno[2,3-*b*]pyridine derivative **8** [42] also occurs (Scheme 3).

Based on these results, we decided to accomplish the process in the *one-pot two-step* format (Scheme 3). In this case, stage I was completed in one hour without heating to avoid the formation of unsubstituted chromeno[2,3-*b*]pyridine derivative **8** [42]. Stage II also took half an hour, but with heating at reflux. At the end of the *two-step* transformation, only 5-*O*-chromeno[2,3-*b*]pyridine **4a** was detected.

**Scheme 4:**
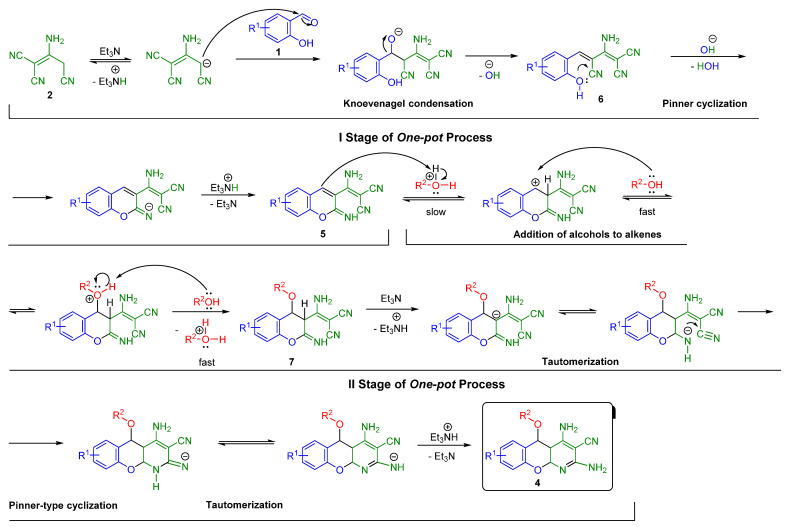
Mechanism of salicylaldehydes **1**, malononitrile dimer **2** and alcohol **3** transformation into 5-*O*-chromeno[2,3-*b*]pyridines **4**.

Based on our earlier results [5,33,38] and data from the literature [43], we propose the following *one-pot two-step* cascade transformation mechanism (Scheme 4). The first stage of the *one-pot* process was a Knoevenagel condensation with the formation of intermediate **6** and the expulsion of a hydroxide anion [44]. This hydroxide anion catalyzed Pinner cyclization of the adduct **6** into the unsaturated intermediate **5**. At the second stage of the *one-pot* process, the addition of alcohol to the double bond of the intermediate compound **5** according to Markovnikov’s rule took place. The resulting intermediate **7** underwent deprotonation, tautomerization, and Pinner-type cyclization. After that, another tautomerization and protonation occurred to yield 5-*O*-chromeno[2,3-*b*]pyridine **4.**

It should be noted that obtained 5-*O*-chromeno[2,3-*b*]pyridines **4** are unstable in DMSO solution. In publication [45], the authors claimed to have synthesized 2,4-diamino-5-ethoxy-9-methoxy-5*H*-chromeno[2,3-*b*]pyridine-3-carbonitrile. Those data, however, are not available, neither in the text of the article nor in the Supplementary Materials [45]. The authors claim that this is due to the fact that the compound **4a** is unstable in DMSO-*d_6_* and in solid form. Indeed, we observed the decomposition of 5-*O*-substituted chromeno[2,3-*b*]pyridines **4** upon standing in DMSO-*d_6_* in a NMR tube at room temperature, while recording NMR spectra. We solved this problem by preparing a fresh solution. In solid form, these compounds **4** turned out to be stable.

In the case of 5-*P* and *5-C*-substituted chromeno[2,3-*b*]pyridines, corresponding CH acid or trialkyl phosphites acted similarly to alcohol, as described in our research (Scheme 3). It should be noted that *5-P*- and 5-*C*-chromeno[2,3-*b*]pyridines were synthesized under the same conditions (in ethanol) without any quantity of 5-alkoxy-5*H*- chromeno[2,3-*b*]pyridine formation. Moreover, 5-*P* and *5-C*-substituted chromeno[2,3-*b*]pyridines [5,33,34,35,36,37,38] were stable in DMSO. This affirms that *5-P*- and 5-*C*-products are thermodynamically more stable, and that 5-alkoxy derivatives are kinetic products.

### 2.3. One-Pot Synthesis of 5-Alkoxy-5H-Chromeno[2,3-b]pyridines ***4a***–***i***

As the mechanism of the reaction had been established, we were interested in optimizing the reaction conditions. Further, transformations of salicylaldehyde **1a**, malononitrile dimer **2** and alcohol **3a** were done in the *one-pot, two-step* format (Scheme 3, Path A, *two-step, one-pot*) to avoid the concurrent formation of byproduct **8** (Scheme 3, Path B). Namely, salicylaldehyde **1a** and malononitrile dimer **2** in alcohol were stirred until the intermediate **5** was precipitated (Stage I), and the reaction mixture was heated at reflux to produce 5-*O*-chromeno[2,3-*b*]pyridines **4** (Stage II). The list of conditions is presented in Table 1.

**Table 1:** Optimization of reaction conditions on the synthesis of **4a** ^1^.

Entry	Catalyst	Cat. Amount (mol%)	Solvent Amount st. II (mL)	Time st. II (h)	Temp. (°C)	Yield (%) ^2^
1	Morpholine	10	20	1	65	80
2	Piperidine	10	20	1	65	82
3	Pyridine	10	20	1	65	71
4	Et_3_N	10	20	1	65	94
5	AcONa	10	20	1	65	48 ^3^
6	NaOH	10	20	1	65	52 ^3^
7	KF	10	20	1	65	29 ^3^
8	Et_3_N	20	20	1	65	95
9	Et_3_N	10	15	1	65	90
10	Et_3_N	10	25	1	65	82
11	Et_3_N	10	20	0.5	65	93
12	Et_3_N	10	20	2	65	94
13	Et_3_N	10	20	0.5	23 (rt)	10 ^3^

^1^ Reaction conditions: salicylaldehyde **1a** (1 mmol), malononitrile dimer **2** (1 mmol), methanol **3a** (1 mmol) were stirred in 10 mL of corresponding alcohol at room temperature for 1 h, then another portion of alcohol was added, and reaction mixture was heated at reflux for time indicated in Table 1. ^2^ Isolated yields. ^3^ NMR data.

The data from Table 1 indicate that amines (Entries 1–4, 8–13) tend to produce higher yields of **4a** than inorganic catalysts. Stage I can be accomplished with any type of catalyst in high yields. It is possible that amines form a transitional intermediate that increases the rate of the nucleophilic addition in Stage II.

The reaction in Stage I is easily catalyzed, and the product precipitates in one hour process without heating. In Stage II, there should be a balance between reagent and product solubility, and the amount of alcohol is important (Entries 8–10). Thus, 10 mL of methanol were used in Stage I, and another 20 mL of methanol were used in Stage II to achieve the best results. According to Entries 8, 11, 12, Stage II finishes in a half hour.

Among the amines used (Entries 1–4), triethylamine (Entry 4) is the best catalyst. This is probably due to its strong basicity. 10% of catalyst is enough for the reaction (Entries 4, 8).

To sum up, Entry 11 is the most optimal condition for this new transformation. To isolate the synthesized compound, when the reaction was finished, the reaction mixture was left at room temperature for 3 h to crystallize the final compound **4** in pure form.

Under the optimal conditions, multicomponent reactions of salicylaldehydes **1a**–**c**, malononitrile dimer **2** and alcohols **3a**–**c** were carried out. 5-Alkoxy-5*H*-chromeno[2,3-*b*]pyridines **4a**–**i** were obtained in 77–93% yields (Table 2).

It should be noted that regardless of the type of alcohol, the highest yields of chromeno[2,3-*b*]pyridine **4a**–**c** were obtained in the reaction with salicylaldehyde **1a** (Table 2). When electron-donor or halogen substituted salicylaldehyde **1** is used, the yields decrease slightly but remain comparable. This is preusmably due to the greater solubility of the intermediate **5** in alcohols in these cases. When alcohol was changed, the highest yields of chromeno[2,3-*b*]pyridines **4** were obtained with methanol **3a** (Table 2). Most likely, this can be explained by steric factors.

### 2.4. 2D-NMR Study of the Structure of Compound ***4d***

**Figure 3:**
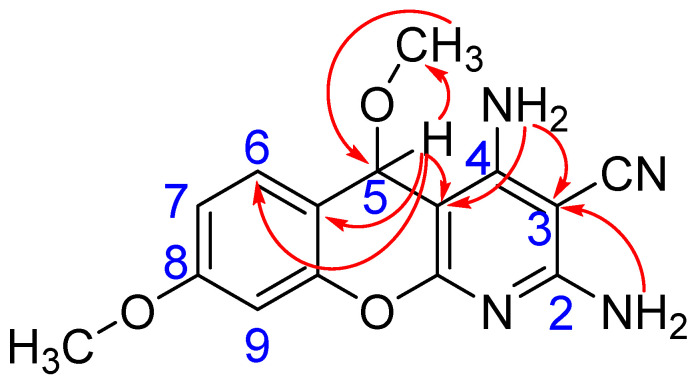
The structure of chromeno[2,3-*b*]pyridine **4d**. Key ^1^H-^13^C-HMBC NMR correlations are shown by arrows.

**Figure 4:**
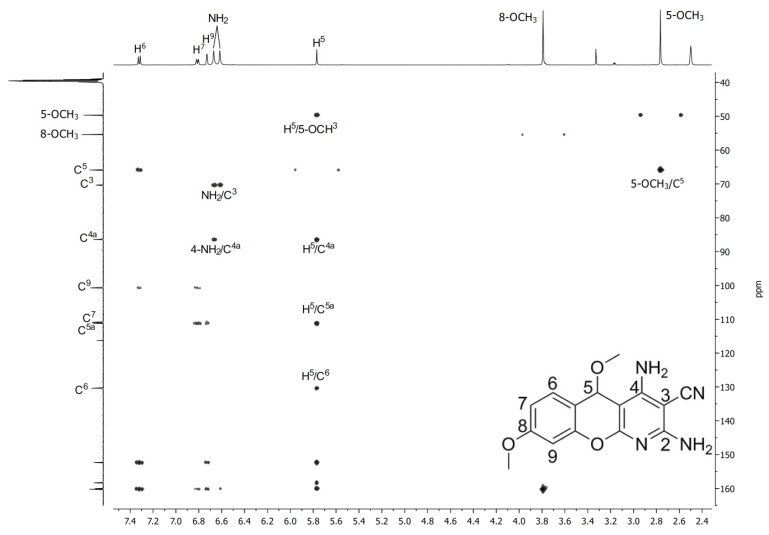
^1^H-^13^C HMBC NMR spectrum of 2,4-diamino-5,8-dimethoxy-5*H*-chromeno[2,3-*b*]pyridine-3-carbonitrile **4d** in DMSO-*d_6_* (400 MHz, 298 K).

The structure of the obtained compounds **4a**–**i** was confirmed by ^1^H and ^13^C NMR data, IR spectroscopy, and mass spectrometry. The structure of the compound **4d** was confirmed by various NMR correlation spectroscopy techniques (Figure 3 and Figure 4).

The assignment of one-dimensional (1D) ^1^H and ^13^C-NMR spectra signals was performed using two-dimensional (2D) NMR experiments such as ^1^H-^13^C HSQC and ^1^H-^13^C HMBC (Figure 4). ^1^H-^13^C HSQC showed two NH_2_-groups which had no cross-peaks in the spectrum. In the HMBC spectrum, the coupling between amino-protons and C^3^ was found (Figure 3 and Figure 4). Additionally, 5-OMe gave the only correlation with C^5^ through three chemical bonds. This is confirmed by the spin interaction from the HMBC spectrum of H^5^ with the carbons of the benzene and pyridine rings (Figure 3 and Figure 4).

The full correlation of signals from one-dimensional ^1^H and ^13^C-NMR spectra with the corresponding atoms of compound **4d** is as follows:

^1^H-NMR (500 MHz, DMSO-*d*_6_) *δ*: 2.77 (s, 3H, 5-OCH_3_), 3.79 (s, 3H, 8-OCH_3_), 5.77 (s, 1H, C(H^5^)), 6.62 (s, 2H, 2-NH_2_), 6.67 (s, 2H, 4-NH_2_), 6.73 (d, ^4^*J* = 2.5 Hz, 1H, C(H^9^) Ar), 6.81 (dd, ^3^*J* = 8.5 Hz, ^4^*J* = 2.5 Hz, 1H, C(H^7^) Ar), 7.32 (d, ^3^*J* = 8.5 Hz, 1H, C(H^6^) Ar) ppm.

^13^C-NMR (126 MHz, DMSO-*d*_6_) *δ*: 49.7 (5-OCH_3_), 55.4 (8-OCH_3_), 65.9 (C^5^), 70.3 (C^3^), 86.5 (C^4a^), 100.7 (C^9^), 110.9 (C^7^), 111.2 (C^5a^), 116.3 (CN), 130.4 (C^6^), 152.3 (C^9a^), 158.4, 160.0 (C^4^, C^1a^), 160.23, 160.24 (C^2^, C^8^) ppm.

Detailed 1D ^1^H and ^13^C-NMR spectra and 2D NMR spectra of the compound **4d** are presented in the Supplementary Materials (Figures S19–S22).

## 3. Materials and Methods

### 3.1. General Information

The solvents and reagents were purchased from commercial sources and used as received. 2-Aminoprop-1-ene-1,1,3-tricarbonitrile (malononitrile dimer) **2** was synthesized from malononitrile according to the literature [46].

All melting points were measured with a Gallenkamp melting-point apparatus (Gallenkamp & Co., Ltd., London, UK) and were uncorrected. ^1^H and ^13^C-NMR spectra were recorded in DMSO-*d_6_* with Bruker AM300 and Bruker AV500 spectrometers (Bruker Corporation, Billerica, MA, USA) at ambient temperature. Chemical shift values are relative to Me_4_Si. Some ^1^H-NMR spectra have underestimated NH_2_ signals integrals. These protons were exchanged with D_2_O (it is present as an impurity in DMSO-*d_6_*). Two-dimensional (2D) NMR spectra were registered with a Bruker AV400 spectrometer (Bruker Corporation, Billerica, MA, USA) at ambient temperature. The IR spectrum was recorded with a Bruker ALPHA-T FT-IR spectrometer (Bruker Corporation, Billerica, MA, USA) in a KBr pellet. MS spectra (EI = 70 eV) were obtained directly with a Kratos MS-30 spectrometer (Kratos Analytical Ltd., Manchester, UK). High-resolution mass spectra (HRMS) were measured on a Bruker micrOTOF II (Bruker Corporation, Billerica, MA, USA) instrument using electrospray ionization (ESI).

### 3.2. One-Pot Synthesis of 5-Alkoxy-5H-Chromeno[2,3-b]pyridines ***4a***–***i***

Salicylaldehyde **1a**–**c** (1 mmol) and malononitrile dimer **2** (1 mmol, 132 mg) were stirred in alcohol **3a**–**c** (10 mL) for 1 h at room temperature. The formation of a thick yellowish precipitate was observed. Another portion of alcohol **3a**–**c** (20 mL) was added to the precipitate with stirring, and the reaction mixture was refluxed for an additional 30 min. After the reaction was completed, the flask was left at room temperature for 3 h. The solid was filtered, washed with well-chilled ethanol/water mixture (1:1, 2 × 2 mL), and dried to isolate pure 5-alkoxy-5*H*-chromeno[2,3-*b*]pyridine **4a**–**i**.

**2,4-Diamino-5-methoxy-5*H*-chromeno[2,3-*b*]pyridine-3-carbonitrile 4a**, (white powder, 250 mg, 93%), mp. 297–298 °C (decomp.) (from MeOH), FTIR (KBr), cm^−1^: 3458, 3345, 3228, 2203, 1624, 1600, 1564, 1408, 1217, 1049. ^1^H-NMR (300 MHz, DMSO-*d_6_*) *δ* 2.82 (s, 3H, OCH_3_), 5.85 (s, 1H, CH), 6.64 (s, 2H, NH_2_), 6.72 (s, 2H, NH_2_), 7.15–7.29 (m, 2H, 2 CH Ar), 7.38–7.50 (m, 2H, 2 CH Ar) ppm. ^13^C-NMR (126 MHz, DMSO-*d_6_*) *δ* 48.7, 66.2, 70.4, 88.5, 115.8, 116.6, 122.1, 124.7, 128.9 (2C), 151.3, 156.7, 159.2, 159.8 ppm. MS (EI, 70 eV) *m/z* (%): 268 [M]^+^ (5), 237 (100), 209 (3), 171 (21), 145 (3), 118 (6), 92 (3), 66 (21), 63 (6), 29 (16). HRMS-ESI: *m/z* [M + H]^+^, calcd for C_14_H_13_N_4_O_2_ 269.1039, found 269.1043.

**2,4-Diamino-5-ethoxy-5*H*-chromeno[2,3-*b*]pyridine-3-carbonitrile 4b**, (white powder, 246 mg, 87%), mp. 294–295 °C (decomp.) (from EtOH), FTIR (KBr), cm^−1^: 3461, 3367, 3226, 2205, 1625, 1601, 1564, 1407, 1216, 1057. ^1^H-NMR (300 MHz, DMSO-*d_6_*) *δ* 0.93 (t, ^3^*J* = 7.0 Hz, 3H, CH_3_), 2.96–3.13 (m, 2H, OCH_2_), 5.83 (s, 1H, CH), 6.60 (s, 2H, NH_2_), 6.69 (s, 2H, NH_2_), 7.12–7.27 (m, 2H, 2 CH Ar), 7.35–7.51 (m, 2H, 2 CH Ar) ppm. ^13^C-NMR (126 MHz, DMSO-*d_6_*) *δ* 15.3, 58.2, 65.6, 70.4, 87.1, 116.3, 119.9, 123.8, 128.8, 129.5, 129.7, 151.1, 158.4, 159.9, 160.3 ppm. MS (EI, 70 eV) *m/z* (%): 282 [M]^+^ (3), 237 (100), 209 (2), 180 (1), 171 (13), 145 (2), 118 (3), 77 (4), 66 (13), 29 (34). HRMS-ESI: *m/z* [M + H]^+^, calcd for C_15_H_15_N_4_O_2_ 283.1195, found 283.1200.

**2,4-Diamino-5-propoxy-5*H*-chromeno[2,3-*b*]pyridine-3-carbonitrile 4c**, (white powder, 264 mg, 89%), mp. 293–294 °C (decomp.) (from *n*-PrOH), FTIR (KBr), cm^−1^: 3464, 3357, 3235, 2198, 1627, 1602, 1564, 1408, 1220, 1069. ^1^H-NMR (300 MHz, DMSO-*d_6_*) *δ* 0.73 (t, ^3^*J* = 7.4 Hz, 3H, CH_3_), 1.42–1.25 (m, 2H, CH_2_), 2.84–3.02 (m, 2H, OCH_2_), 5.89 (s, 1H, CH), 6.62 (s, 2H, NH_2_), 6.69 (s, 2H, NH_2_), 7.13–7.28 (m, 2H, 2 CH Ar), 7.37–7.51 (m, 2H, 2 CH Ar) ppm. ^13^C-NMR (75 MHz, DMSO-*d_6_*) *δ* 10.6, 22.6, 64.1, 65.6, 70.3, 86.9, 116.2, 119.7, 123.8, 128.8, 129.5, 129.7, 151.1, 158.4, 159.8, 160.3 ppm. MS (EI, 70 eV) *m/z* (%): 296 [M]^+^ (3), 253 (2), 237 (100), 209 (2), 171 (14), 145 (2), 118 (3), 77 (2), 66 (9), 29 (16). HRMS-ESI: *m/z* [M + H]^+^, calcd for C_16_H_17_N_4_O_2_ 297.1346, found 297.1352.

**2,4-Diamino-5,8-dimethoxy-5*H*-chromeno[2,3-*b*]pyridine-3-carbonitrile 4d**, (yellowish powder, 239 mg, 80%), mp. 282–283 °C (decomp.) (from MeOH), FTIR (KBr), cm^−1^: 3464, 3354, 3234, 2197, 1629, 1600, 1559, 1403, 1173, 1048. ^1^H-NMR (300 MHz, DMSO-*d_6_*) *δ* 2.79 (s, 3H, OCH_3_), 3.81 (s, 3H, CH_3_O-Ar), 5.79 (s, 1H, CH), 6.62 (s, 2H, NH_2_), 6.67 (s, 2H, NH_2_), 6.75 (s, 1H, 1 CH Ar), 6.83 (d, ^3^*J* = 8.3 Hz, 1H, 1 CH Ar), 7.34 (d, ^3^*J* = 8.3 Hz, 1H, 1 CH Ar) ppm. ^13^C-NMR (75 MHz, DMSO-*d_6_*) *δ* 49.6, 55.4, 65.9, 70.4, 86.5, 100.7, 110.9, 111.2, 116.2, 130.4, 152.3, 158.4, 160.0, 160.2 (2C) ppm. MS (EI, 70 eV) *m/z* (%): 298 [M]^+^ (2), 267 (100), 224 (19), 195 (4), 170 (2), 133 (4), 114 (1), 77 (2), 66 (5), 15 (23). HRMS-ESI: *m/z* [M + H]^+^, calcd for C_15_H_15_N_4_O_3_ 299.1144, found 299.1148.

**2,4-Diamino-5-ethoxy-8-methoxy-5*H*-chromeno[2,3-*b*]pyridine-3-carbonitrile 4e**, (yellowish powder, 244 mg, 78%,), mp. 280–281 °C (decomp.) (from EtOH), FTIR (KBr), cm^−1^: 3472, 3309, 3201, 2200, 1629, 1600, 1566, 1407, 1205, 1050. ^1^H-NMR (300 MHz, DMSO-*d_6_*) *δ* 0.95 (t, ^3^*J* = 6.9 Hz, 3H, CH_3_), 3.02 (q, ^3^*J* = 6.9 Hz, 2H, OCH_2_), 3.81 (s, 3H, CH_3_O-Ar), 5.79 (s, 1H, CH), 6.60 (s, 2H, NH_2_), 6.66 (s, 2H, NH_2_), 6.74 (d, ^4^*J* = 2.1 Hz, 1H, 1 CH Ar), 6.82 (dd, ^3^*J* = 8.5 Hz, ^4^*J* = 2.1 Hz, 1H, 1 CH Ar), 7.35 (d, ^3^*J* = 8.5 Hz, 1H, 1 CH Ar) ppm. ^13^C-NMR (75 MHz, DMSO-*d_6_*) *δ* 15.3, 55.4, 57.7, 65.3, 70.4, 87.2, 100.8, 110.8, 112.0, 116.3, 130.3, 152.0, 152.1, 158.3, 159.6, 160.2 ppm. MS (EI, 70 eV) *m/z* (%): 312 [M]^+^ (2), 267 (100), 224 (26), 195 (4), 170 (2), 141 (2), 133 (7), 77 (3), 66 (8), 29 (47). HRMS-ESI: *m/z* [M + H]^+^, calcd for C_16_H_17_N_4_O_3_ 313.1301, found 313.1307.

**2,4-Diamino-8-methoxy-5-propoxy-5*H*-chromeno[2,3-*b*]pyridine-3-carbonitrile 4f**, (yellowish powder, 258 mg, 79%), mp. 284–285 °C (decomp.) (from *n*-PrOH), FTIR (KBr), cm^−1^: 3465, 3358, 3237, 2198, 1628, 1601, 1563, 1408, 1175, 1071. ^1^H-NMR (300 MHz, DMSO-*d_6_*) *δ* 0.73 (t, ^3^*J* = 7.4 Hz, 3H, CH_3_), 1.25–1.41 (m, 2H, CH_2_), 2.80–2.96 (m, 2H, OCH_2_), 3.81 (s, 3H, CH_3_O-Ar), 5.83 (s, 1H, CH), 6.61 (s, 2H, NH_2_), 6.65 (s, 2H, NH_2_), 6.73 (d, ^4^*J* = 2.1 Hz, 1H, 1 CH Ar), 6.82 (dd, ^3^*J* = 8.5 Hz, ^4^*J* = 2.1 Hz, 1H, 1 CH Ar), 7.35 (d, ^3^*J* = 8.5 Hz, 1H, 1 CH Ar) ppm. ^13^C-NMR (75 MHz, DMSO-*d_6_*) *δ* 10.6, 22.6, 55.4, 62.4, 65.3, 70.4, 100.7, 110.9, 111.8, 115.7, 116.3, 130.3, 152.1, 157.0, 157.6, 159.6, 160.2 ppm. MS (EI, 70 eV) *m/z* (%): 326 [M]^+^ (1), 267 (100), 252 (6), 224 (12), 195 (2), 170 (1), 134 (3), 104 (1), 66 (1), 31 (3). HRMS-ESI: *m/z* [M + H]^+^, calcd for C_17_H_19_N_4_O_3_ 327.1457, found 327.1461.

**2,4-Diamino-7-chloro-5-methoxy-5*H*-chromeno[2,3-*b*]pyridine-3-carbonitrile 4g**, (white powder, 257 mg, 85%), mp. 306–307 °C (decomp.) (from MeOH), FTIR (KBr), cm^−1^: 3460, 3352, 3229, 2201, 1625, 1598, 1561, 1423, 1222, 1051. ^1^H-NMR (300 MHz, DMSO-*d_6_*) *δ* 2.80 (s, 3H, OCH_3_), 5.81 (s, 1H, CH), 6.66 (s, 2H, NH_2_ exch. with D_2_O), 6.72 (s, 2H, NH_2_ exch. with D_2_O), 7.23 (d, ^3^*J* = 8.6 Hz, 1H, 1 CH Ar), 7.40–7.50 (m, 2H, 2 CH Ar) ppm. ^13^C-NMR (75 MHz, DMSO-*d_6_*) *δ* 50.2, 66.0, 70.4, 85.7, 116.2, 118.5, 121.1, 127.5, 128.9, 129.9, 150.2, 158.4, 159.8, 160.4 ppm. MS (EI, 70 eV) *m/z* (%): 304 [M]^+^ (^37^Cl, 1), 302 [M]^+^ (^35^Cl, 4), 273 (^37^Cl, 32), 271 (^35^Cl, 100), 236 (3), 207 (^37^Cl, 4), 205 (^35^Cl, 12), 179 (2), 136 (5), 114 (3), 75 (4), 66 (23), 29 (17). HRMS-ESI: *m/z* [M + H]^+^, calcd for C_14_H_12_ClN_4_O_2_ 305.0619 (^37^Cl), 303.0649 (^35^Cl), found 305.0622 (^37^Cl), 303.0653 (^35^Cl).

**2,4-Diamino-7-chloro-5-ethoxy-5*H*-chromeno[2,3-*b*]pyridine-3-carbonitrile 4h**, (yellowish powder, 244 mg, 77%), mp. 303–304 °C (decomp.) (from EtOH), FTIR (KBr), cm^−1^: 3467, 3360, 3223, 2197, 1621, 1589, 1562, 1399, 1220, 1051. ^1^H-NMR (300 MHz, DMSO-*d_6_*) *δ* 0.94 (t, ^3^*J* = 6.9 Hz, 3H, CH_3_), 2.95–3.12 (m, 2H, OCH_2_), 5.81 (s, 1H, CH), 6.65 (s, 2H, NH_2_ exch. with D_2_O), 6.71 (s, 2H, NH_2_ exch. with D_2_O), 7.22 (d, ^3^*J* = 9.0 Hz, 1H, 1 CH Ar), 7.41–7.51 (m, 2H, 2 CH Ar) ppm. ^13^C-NMR (75 MHz, DMSO-*d_6_*) *δ* 15.3, 58.4, 65.5, 70.5, 86.5, 116.2, 118.5, 121.8, 127.5, 128.7, 129.7, 150.0, 158.4, 159.7, 160.3 ppm. MS (EI, 70 eV) *m/z* (%): 318 [M]^+^ (^37^Cl, 1), 316 [M]^+^ (^35^Cl, 3), 273 (^37^Cl, 33), 271 (^35^Cl, 100), 236 (3), 207 (^37^Cl, 3), 205 (^35^Cl, 9), 179 (1), 152 (1), 136 (2), 77 (1), 66 (7), 29 (16). HRMS-ESI: *m/z* [M + H]^+^, calcd for C_15_H_14_ClN_4_O_2_ 319.0771 (^37^Cl), 317.0800 (^35^Cl), found 319.0773 (^37^Cl), 317.0802 (^35^Cl).

**2,4-Diamino-7-chloro-5-propoxy-5*H*-chromeno[2,3-*b*]pyridine-3-carbonitrile 4i**, (yellowish powder, 265 mg, 80%), mp. 304–305 °C (decomp.) (from *n*-PrOH), IR spectrum (*ν*, cm^−1^): 3470, 3361, 3227, 2198, 1621, 1598, 1562, 1399, 1222, 1068. ^1^H-NMR (300 MHz, DMSO-*d_6_*) *δ* 0.71 (t, ^3^*J* = 7.4 Hz, 3H, CH_3_), 1.25–1.40 (m, 2H, CH_2_), 2.81–2.98 (m, 2H, OCH_2_), 5.85 (s, 1H, CH), 6.65 (s, 2H, NH_2_ exch. with D_2_O), 6.70 (s, 2H, NH_2_ exch. with D_2_O), 7.21 (d, ^3^*J* = 8.5 Hz, 1H, 1 CH Ar), 7.42–7.48 (m, 2H, 2 CH Ar) ppm. ^13^C-NMR (75 MHz, DMSO-*d_6_*) *δ* 10.6, 22.6, 64.3, 65.5, 70.4, 86.3, 116.2, 118.5, 121.7, 127.5, 128.8, 129.8, 150.0, 158.4, 159.6, 160.3 ppm. MS (EI, 70 eV) *m/z* (%): 332 [M]^+^ (^37^Cl, 1), 330 [M]^+^ (^35^Cl, 3), 273 (^37^Cl, 33), 271 (^35^Cl, 100), 243 (1), 207 (^37^Cl, 4), 205 (^35^Cl, 11), 152 (2), 136 (5), 92 (1), 66 (14), 41 (11), 29 (33). HRMS-ESI: *m/z* [M + H]^+^, calcd for C_16_H_16_ClN_4_O_2_ 333.0932 (^37^Cl), 331.0962 (^35^Cl), found 333.0935 (^37^Cl), 331.0964 (^35^Cl).

## 4. Conclusions

To conclude, the synthesis of 5-*O*-substituted 5*H*-chromeno[2,3-*b*]pyridines differs from that of 5-*P*- and 5-*C*-substituted derivatives. The nucleophile, which is supposed to interact with the intermediate, determines the final structure.

In this case, the solvent (alcohol) acted as a nucleophile; it interacted with the intermediate formed from salicylaldehyde and malononitrile dimer to form 5-*O*-substituted 5*H*-chromeno[2,3-*b*]pyridines. This *two-step, one-pot* transformation extends the *PASE* approach with ‘component economy’ as alcohol is used both as a solvent and a reactant (solvent-involved reaction).

The mechanistic studies revealed the possibility of concurrent processes. These processes tend to form more thermodynamically stable products. Alcohol, as a weak nucleophile, forms the kinetic product, which is unstable in solution.

The mechanistic studies aided in optimizing reaction conditions. Thus, the *one-pot, two-step* transformation of salicylaldehydes, malononitrile dimer and alcohol proceeds efficiently and quickly with the formation of 5-*O*-substituted 5*H*-chromeno[2,3-*b*]pyridines in high yields of 77–93%. It is easy to isolate the final compounds directly from the reaction mixture.

2D NMR spectroscopy confirmed the proposed structure of synthesized 5*H*-chromeno[2,3-*b*]pyridines.

## Figures and Tables

**Table 2:** *One-pot* reaction of salicylaldehydes **1a**–**c**, malononitrile dimer **2** and alcohols **3a**–**c** ^1^.

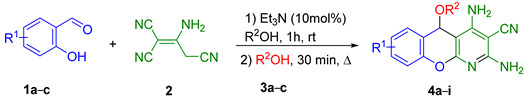
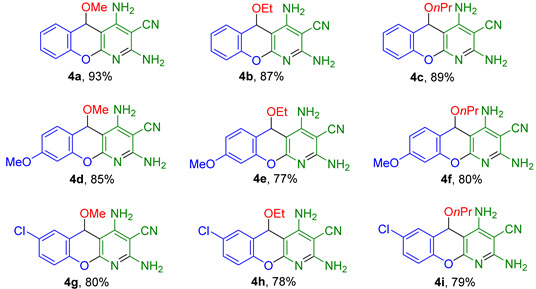

^1^ Reaction conditions: salicylaldehyde **1a**–**c** (1 mmol), malononitrile dimer **2** (1 mmol), and triethylamine (0.1 mmol) were stirred in 10 mL of corresponding alcohol **3a**–**c** for 1 h. Then, another portion of alcohol **3a**–**c** (20 mL) was added, and reaction mixture was heated at reflux for 30 min. Isolated yields.

## Data Availability

Data is contained within the article or Supplementary Material.

## Supplementary Materials

The following supporting information can be downloaded at: https://www.mdpi.com/article/10.3390/molecules28010064/s1, Figures S1–S18: ^1^H and ^13^C Spectra of synthesized compounds **4a–i**, Figures S19 and S20: Detailed 1D-NMR Spectra of **4d**, Figures S21 and S22: 2D-NMR Spectra of **4d**.

## Sample Availability

Samples of the compounds **4a–i** are available from the authors.
