# Supplementary material for: One-Pot Solvent-Involved Synthesis of 5-O-Substituted 5H-Chromeno[2,3-b]pyridines
Source: Molecules. 2022 Dec 21;28(1):64. doi: 10.3390/molecules28010064 (PMC9822245; doi:10.3390/molecules28010064)

Supplementary Material:

One-pot solvent-participated synthesis of 5-*O*-substituted 5*H*-chromeno[2,3-*b*]pyridines

Yuliya E. Ryzhkova, Fedor V. Ryzhkov, Michail N. Elinson*, Oleg I. Maslov, Artem N. Fakhrutdinov

N. D. Zelinsky Institute of Organic Chemistry, Russian Academy of Sciences, 47 Leninsky Pr., Moscow 119991, Russian Federation. E-mail: elinson@ioc.ac.ru

Table of Contents

1. The copies of ^{1}H NMR and ^{13}C NMR spectra for compounds **4a-i**.. S2

2. Detailed 1D ^{1}H and ^{13}C NMR spectra for compound **4d**.. S11

3. 2D NMR data for compound **4d**.. S12

1. The copies of ^{1}H NMR and ^{13}C NMR spectra for compounds 4a-i

Figure S1. ^{1}H NMR spectrum of 2,4-diamino-5-methoxy-5*H*-chromeno[2,3-*b*]pyridine-3-carbonitrile **4a** in DMSO-d_6.

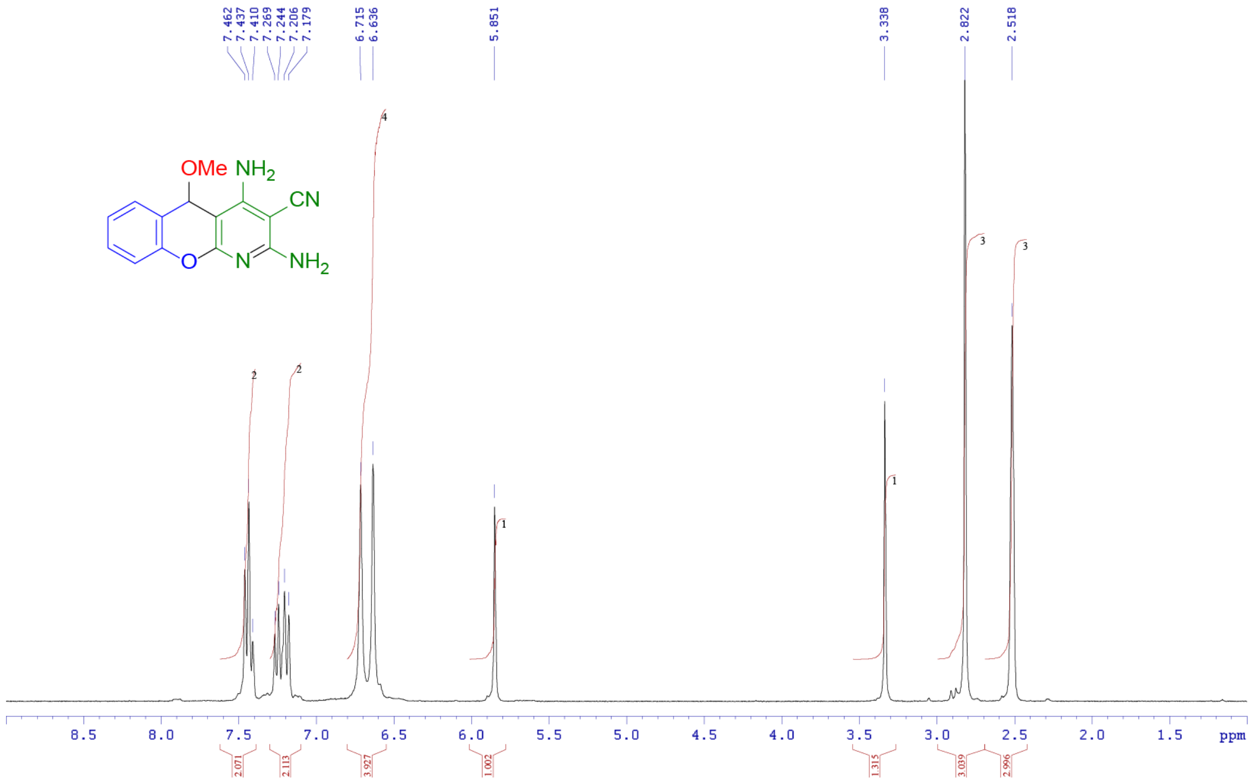

Figure S2. ^{13}C NMR spectrum of 2,4-diamino-5-methoxy-5*H*-chromeno[2,3-*b*]pyridine-3-carbonitrile **4a** in DMSO-d_6.

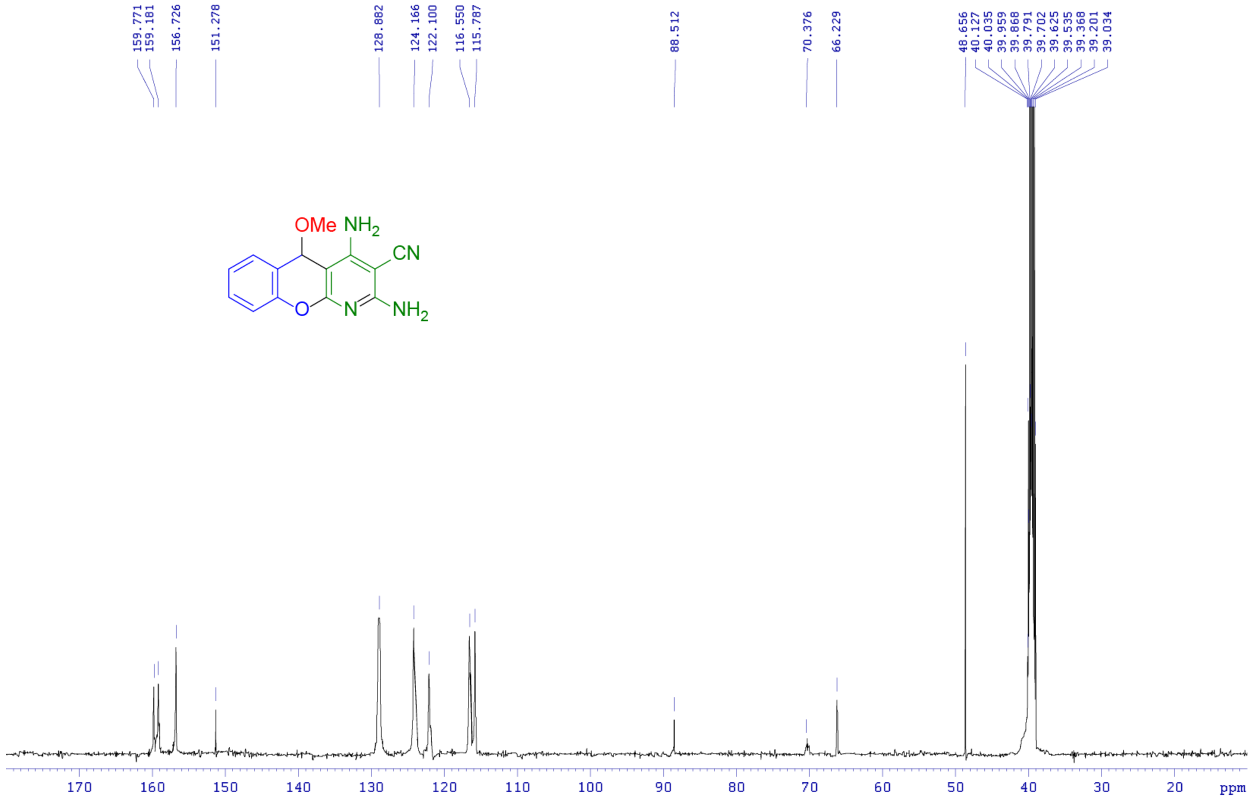

Figure S3. ^{1}H NMR spectrum of 2,4-diamino-5-ethoxy-5*H*-chromeno[2,3-*b*]pyridine-3-carbonitrile **4b** in DMSO-d_6.

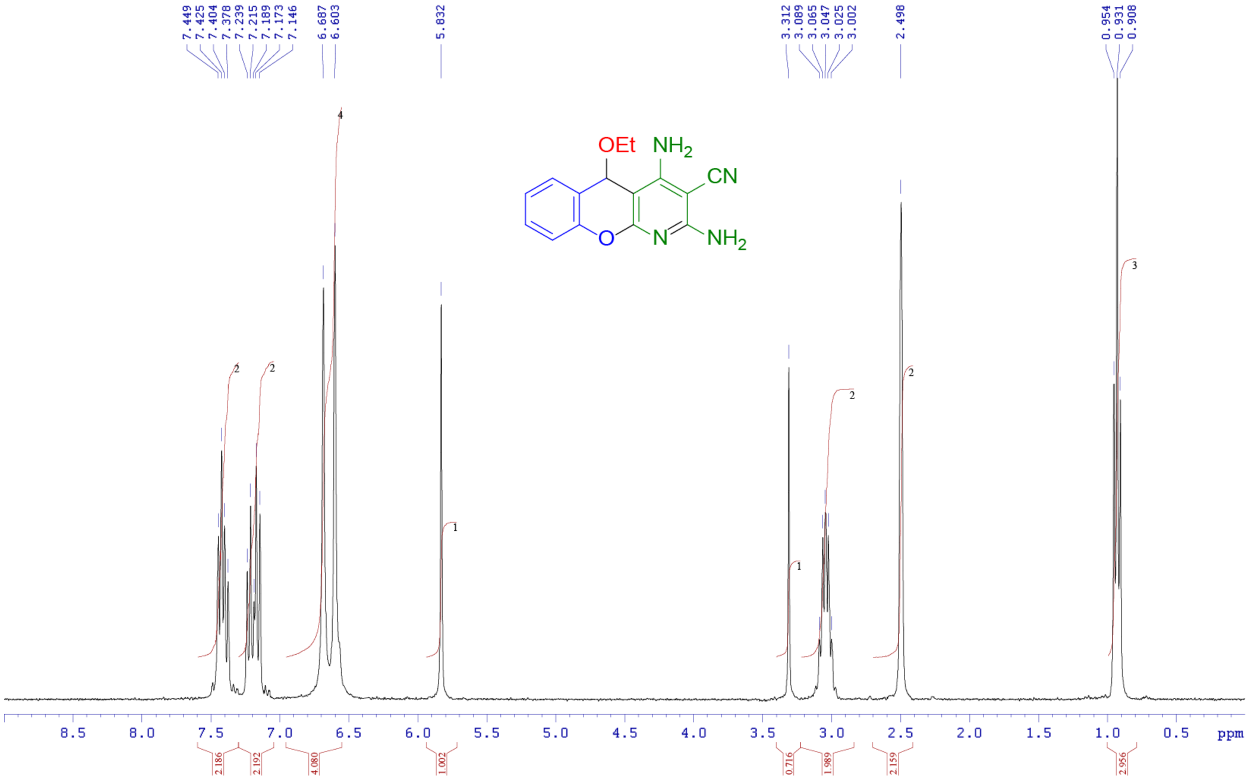

Figure S4. ^{13}C NMR spectrum of 2,4-diamino-5-ethoxy-5*H*-chromeno[2,3-*b*]pyridine-3-carbonitrile **4b** in DMSO-d_6.

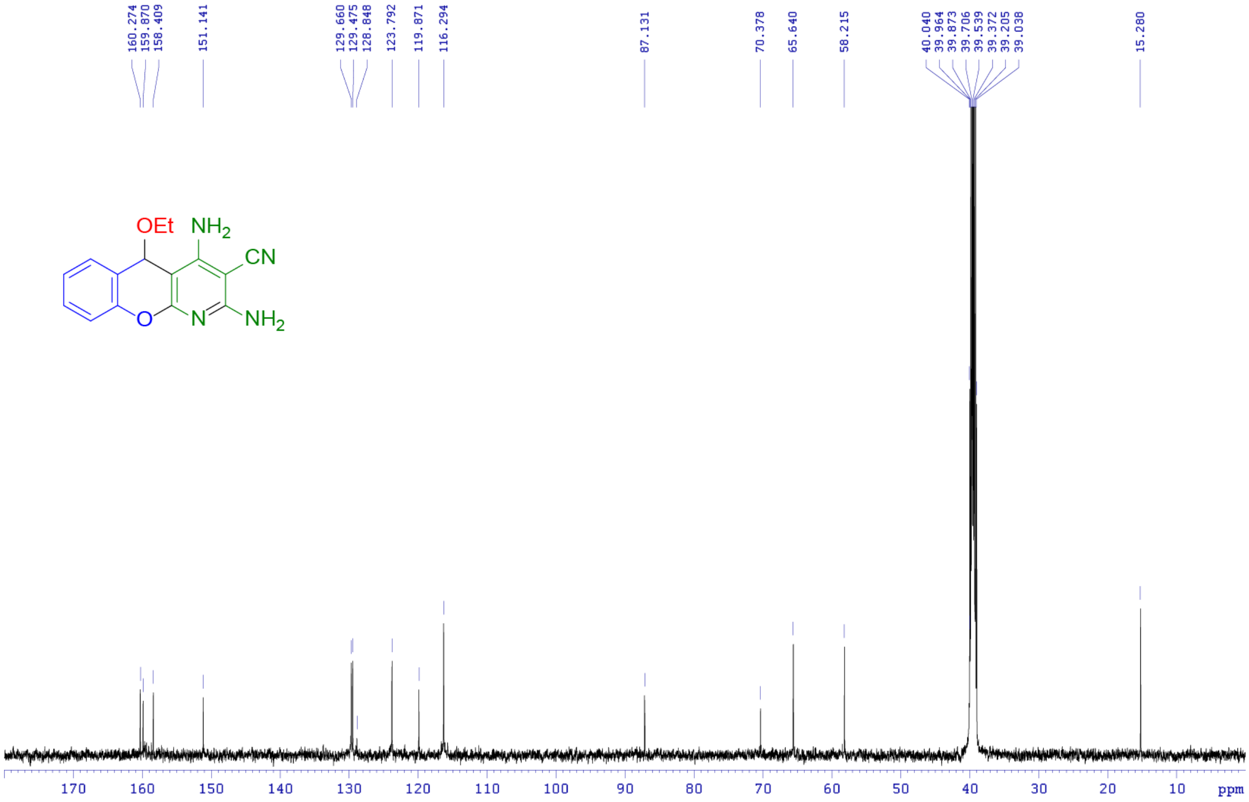

Figure S5. ^{1}H NMR spectrum of 2,4-diamino-5-propoxy-5*H*-chromeno[2,3-*b*]pyridine-3-carbonitrile **4c** in DMSO-*d*$_6$.

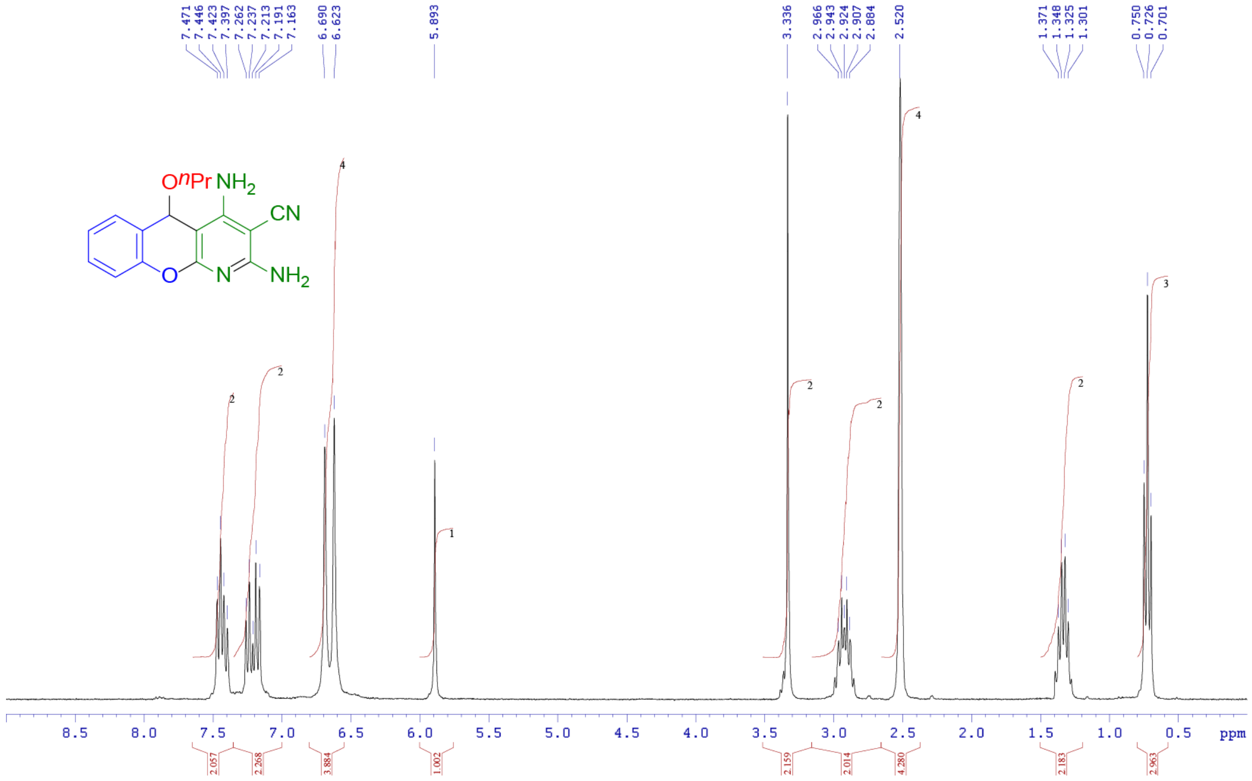

Figure S6. ^{13}C NMR spectrum of 2,4-diamino-5-propoxy-5*H*-chromeno[2,3-*b*]pyridine-3-carbonitrile **4c** in DMSO-*d*$_6$.

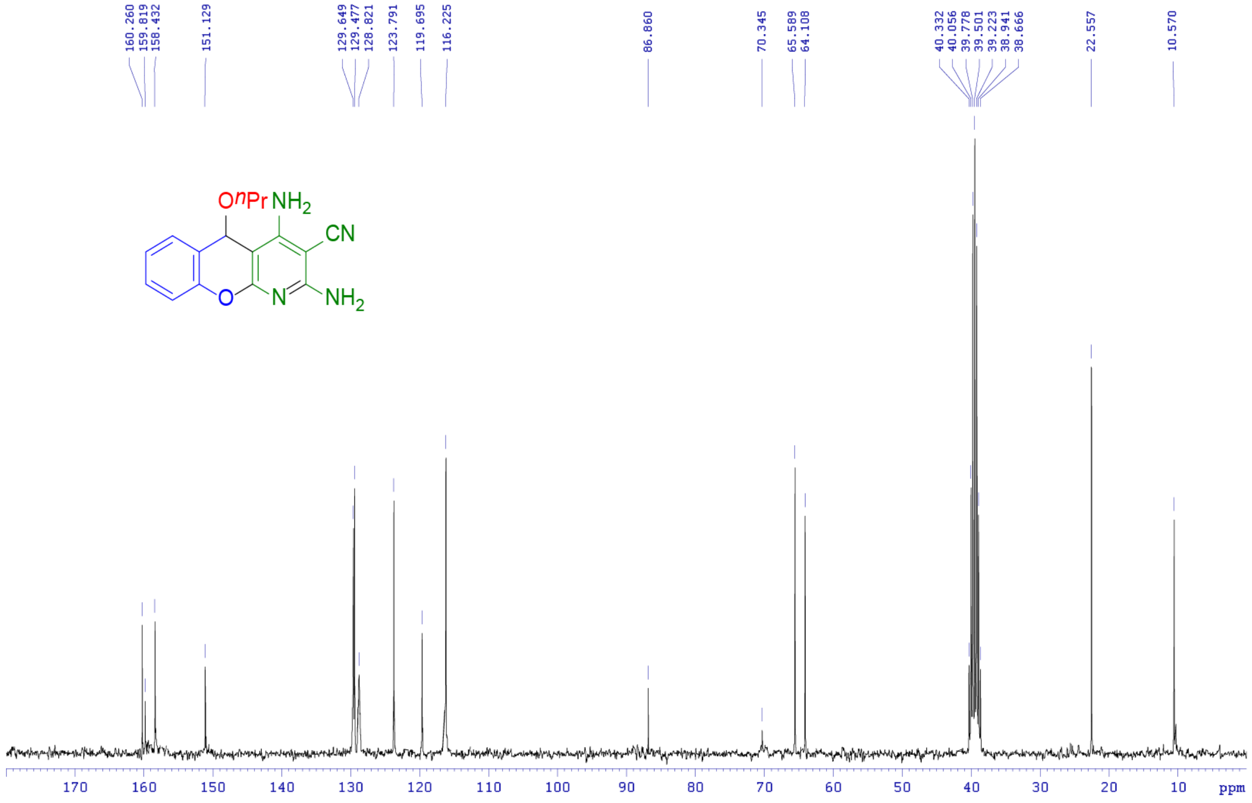

Figure S7. ^{1}H NMR spectrum of 2,4-diamino-5,8-dimethoxy-5*H*-chromeno[2,3-*b*]pyridine-3-carbonitrile **4d** in DMSO-*d*$_6$.

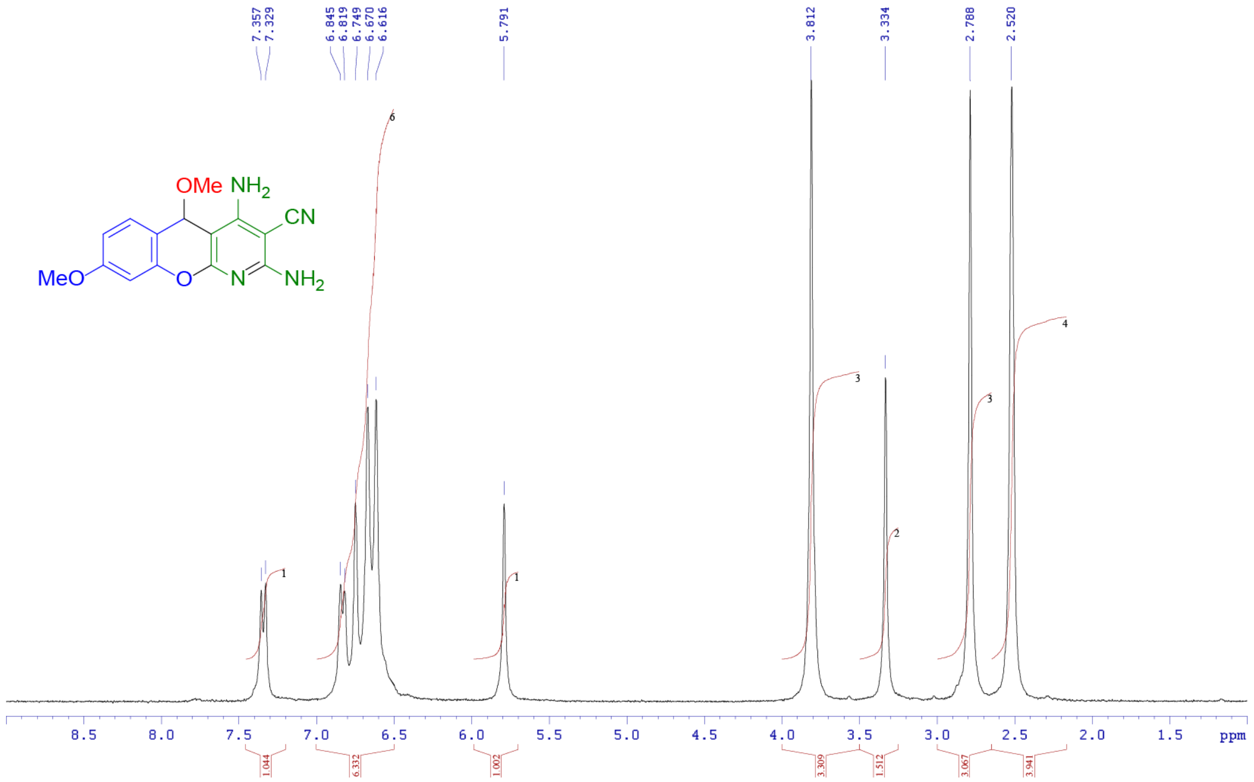

Figure S8. ^{13}C NMR spectrum of 2,4-diamino-5,8-dimethoxy-5*H*-chromeno[2,3-*b*]pyridine-3-carbonitrile **4d** in DMSO-*d*$_6$.

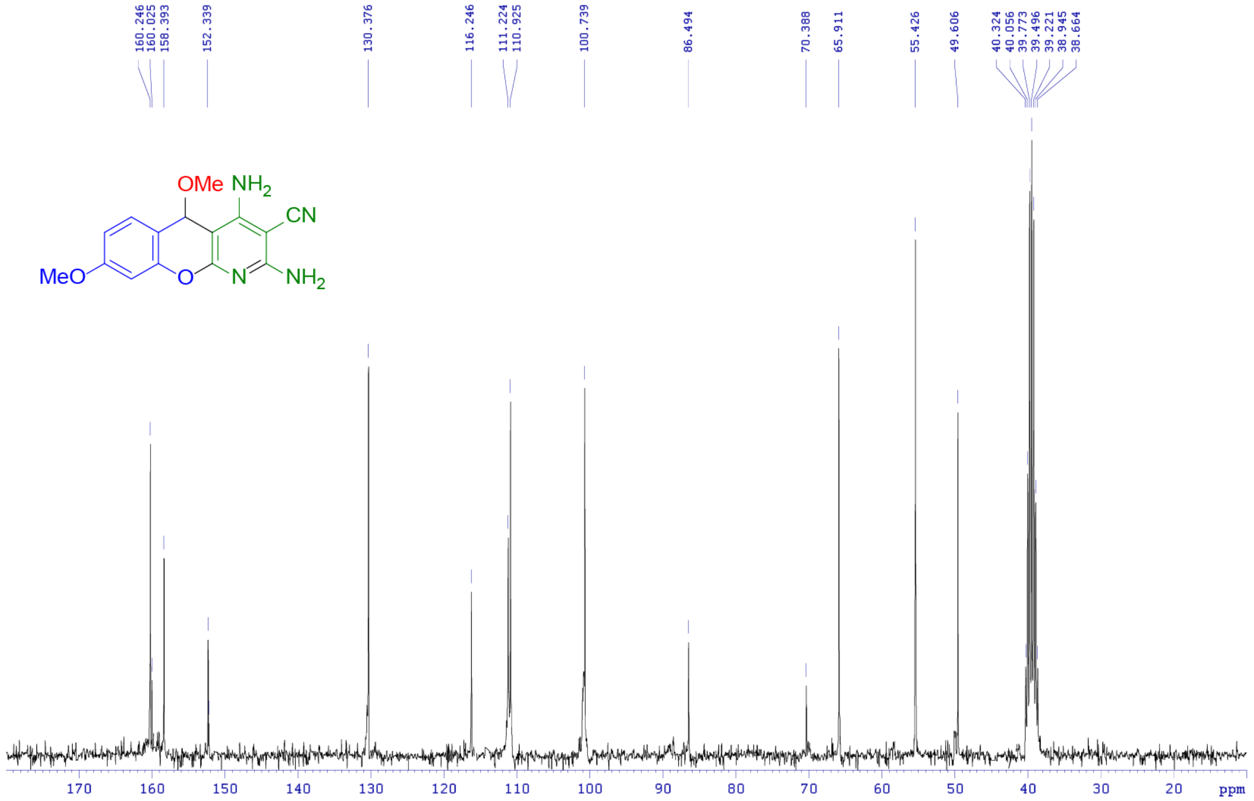

Figure S9. ^{1}H NMR spectrum of 2,4-diamino-5-ethoxy-8-methoxy-5*H*-chromeno[2,3-*b*]-pyridine-3-carbonitrile **4e** in DMSO-*d*6.

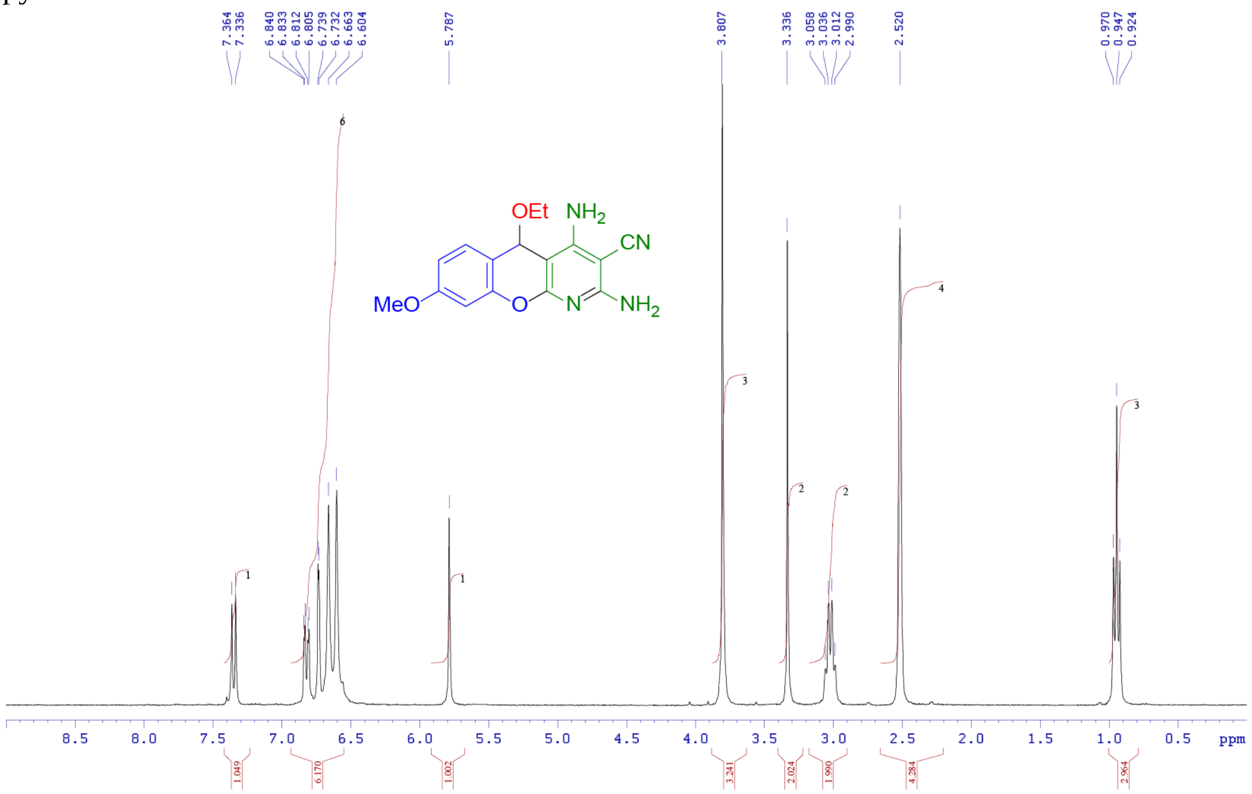

Figure S10. ^{13}C NMR spectrum of 2,4-diamino-5-ethoxy-8-methoxy-5*H*-chromeno[2,3-*b*]-pyridine-3-carbonitrile **4e** in DMSO-*d*6.

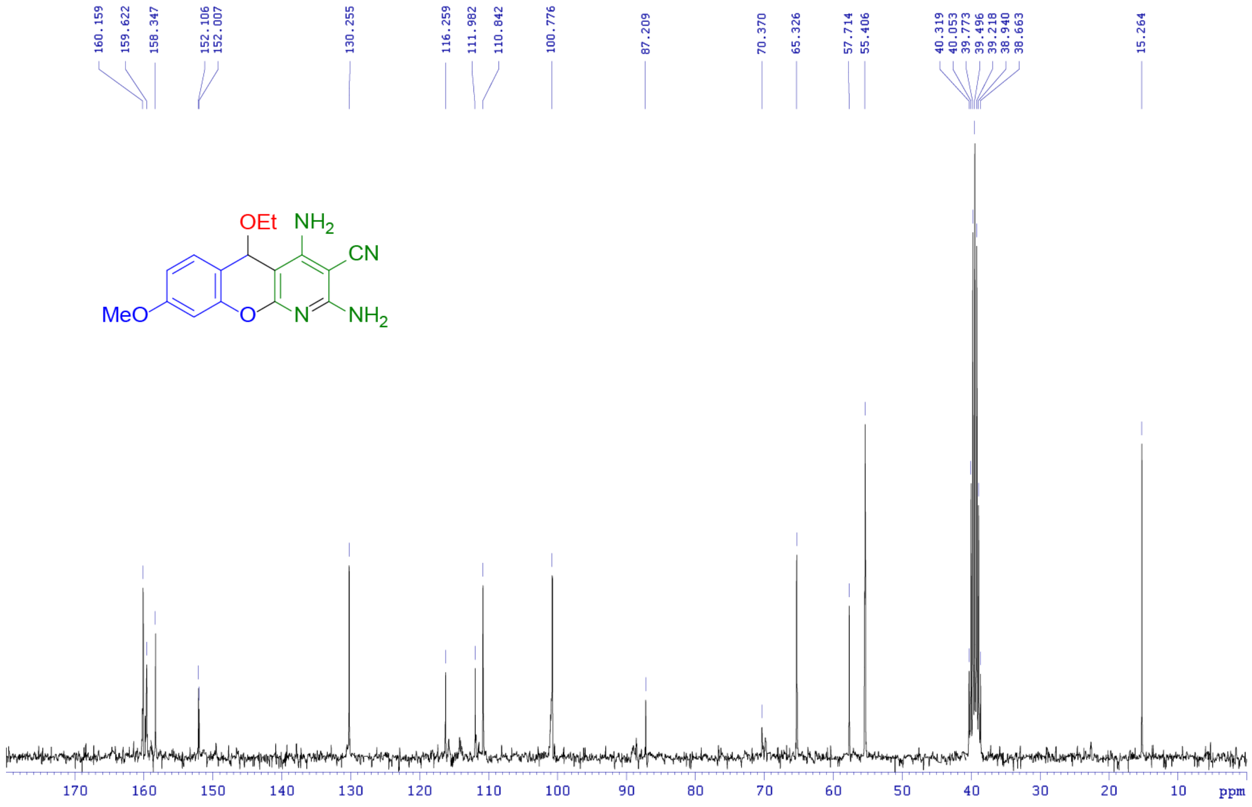

Figure S11. ^{1}H NMR spectrum of 2,4-diamino-8-methoxy-5-propoxy-5*H*-chromeno[2,3-*b*]-pyridine-3-carbonitrile **4f** in DMSO-*d*$_6$.

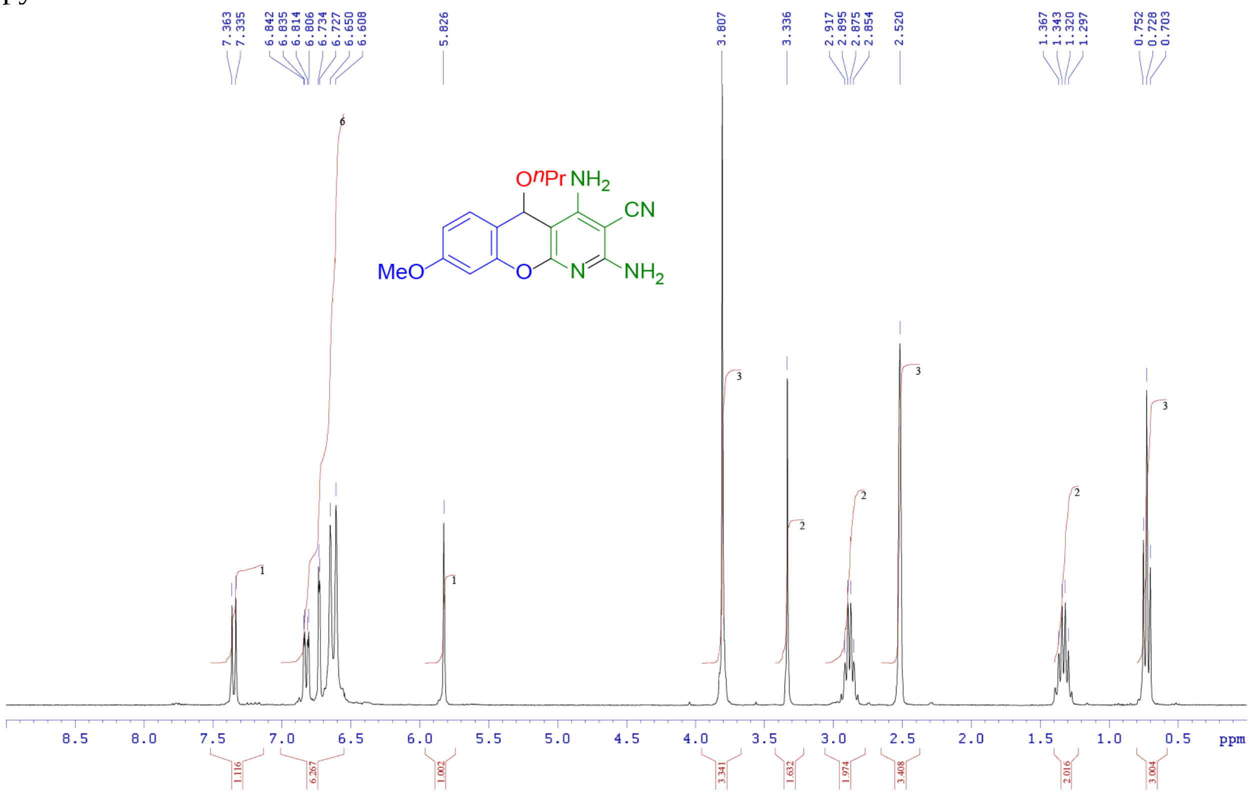

Figure S12. ^{13}C NMR spectrum of 2,4-diamino-8-methoxy-5-propoxy-5*H*-chromeno[2,3-*b*]-pyridine-3-carbonitrile **4f** in DMSO-*d*$_6$.

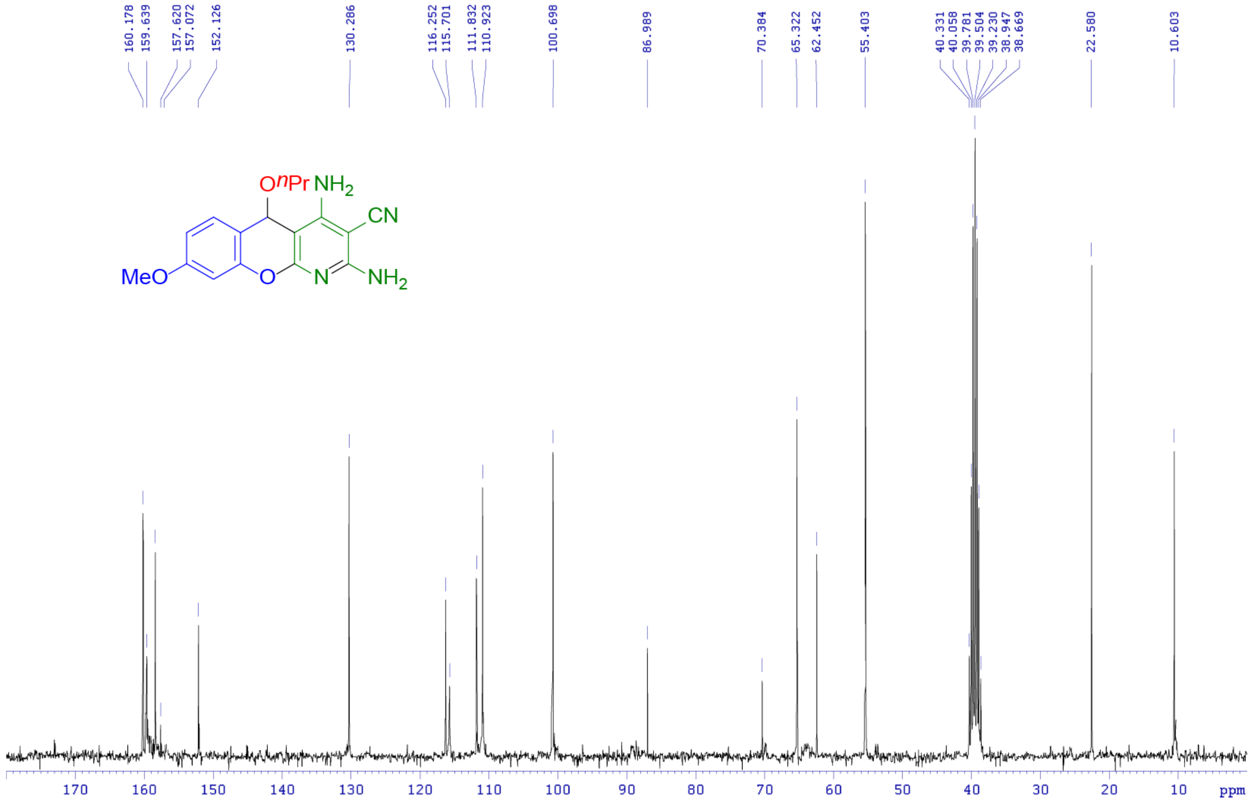

Figure S13. ^{1}H NMR spectrum of 2,4-diamino-7-chloro-5-methoxy-5*H*-chromeno[2,3-*b*]-pyridine-3-carbonitrile **4g** in DMSO-d_6.

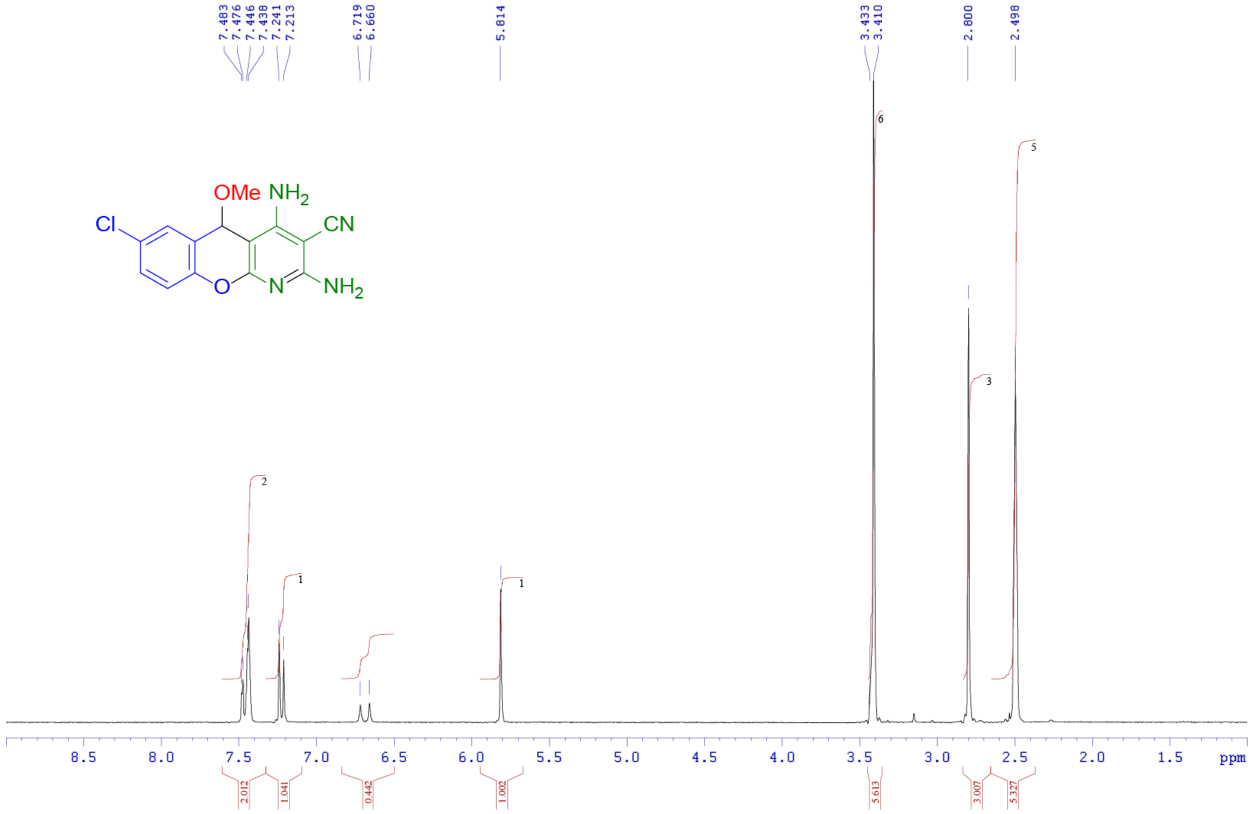

Figure S14. ^{13}C NMR spectrum of 2,4-diamino-7-chloro-5-methoxy-5*H*-chromeno[2,3-*b*]pyridine-3-carbonitrile **4g** in DMSO-d_6.

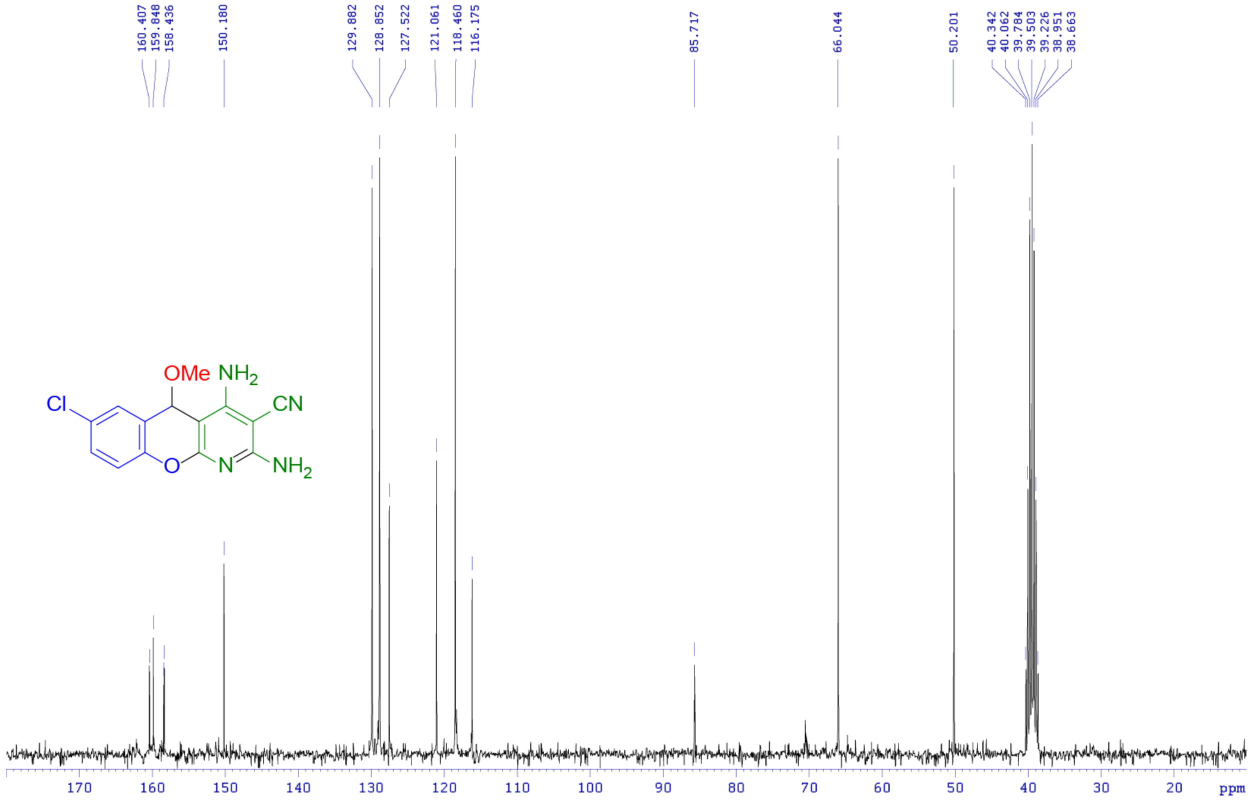

Figure S15. ^{1}H NMR spectrum of 2,4-diamino-7-chloro-5-ethoxy-5*H*-chromeno[2,3-*b*]pyridine-3-carbonitrile **4h** in DMSO-*d*6.

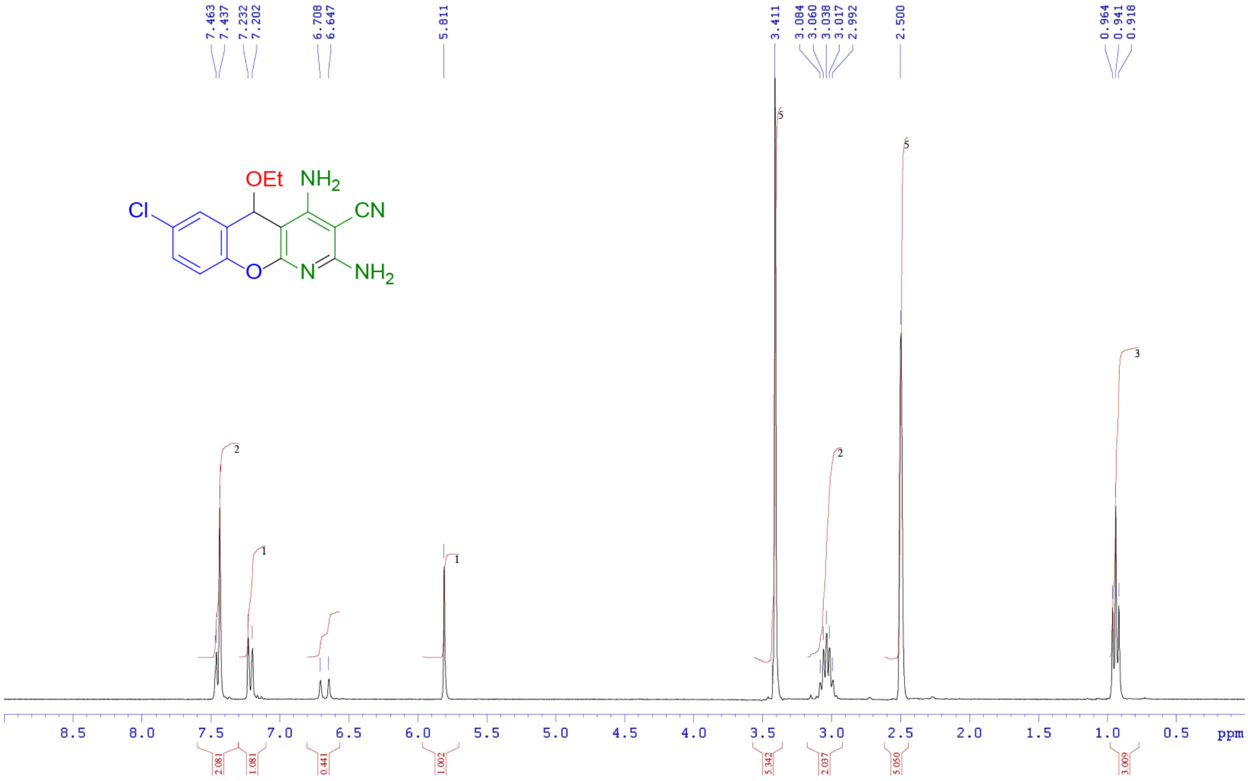

Figure S16. ^{13}C NMR spectrum of 2,4-diamino-7-chloro-5-ethoxy-5*H*-chromeno[2,3-*b*]pyridine-3-carbonitrile **4h** in DMSO-*d*6.

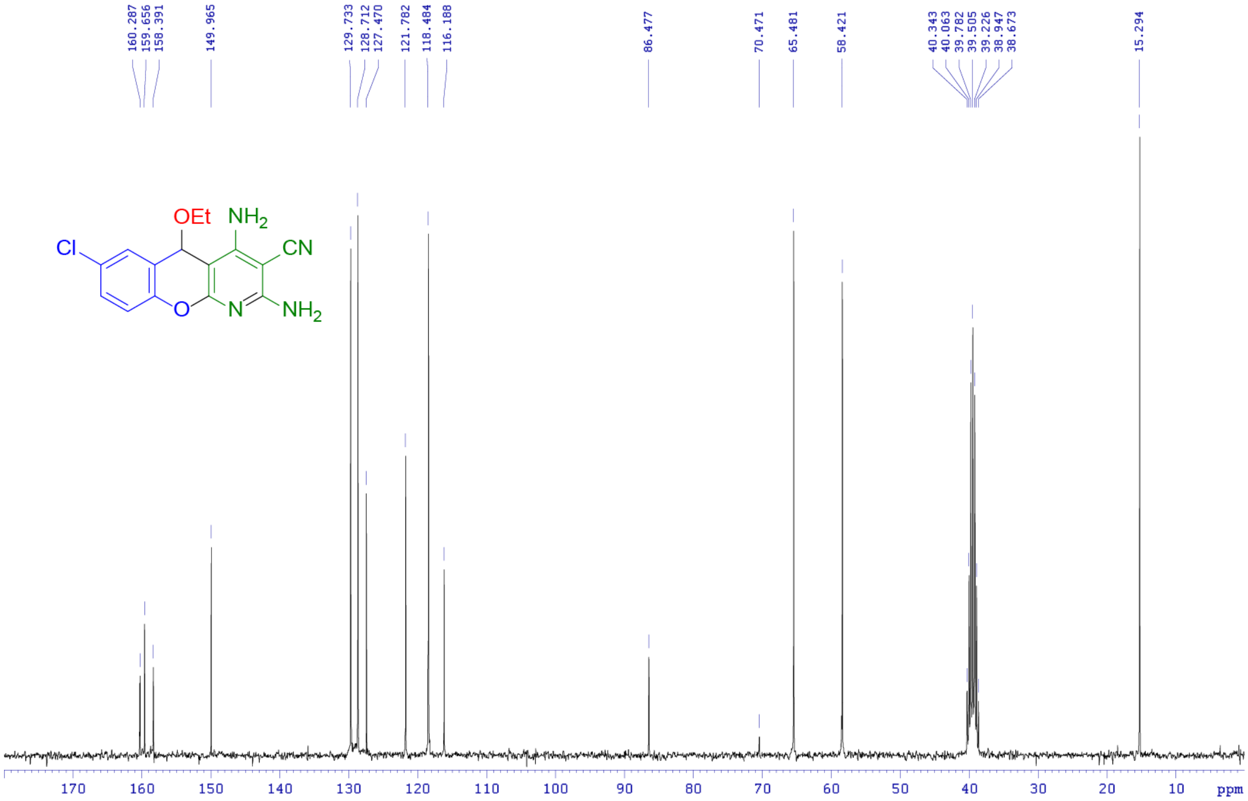

Figure S17. ^{1}H NMR spectrum of 2,4-diamino-7-chloro-5-ethoxy-5*H*-chromeno[2,3-*b*]pyridine-3-carbonitrile **4i** in DMSO-*d*6.

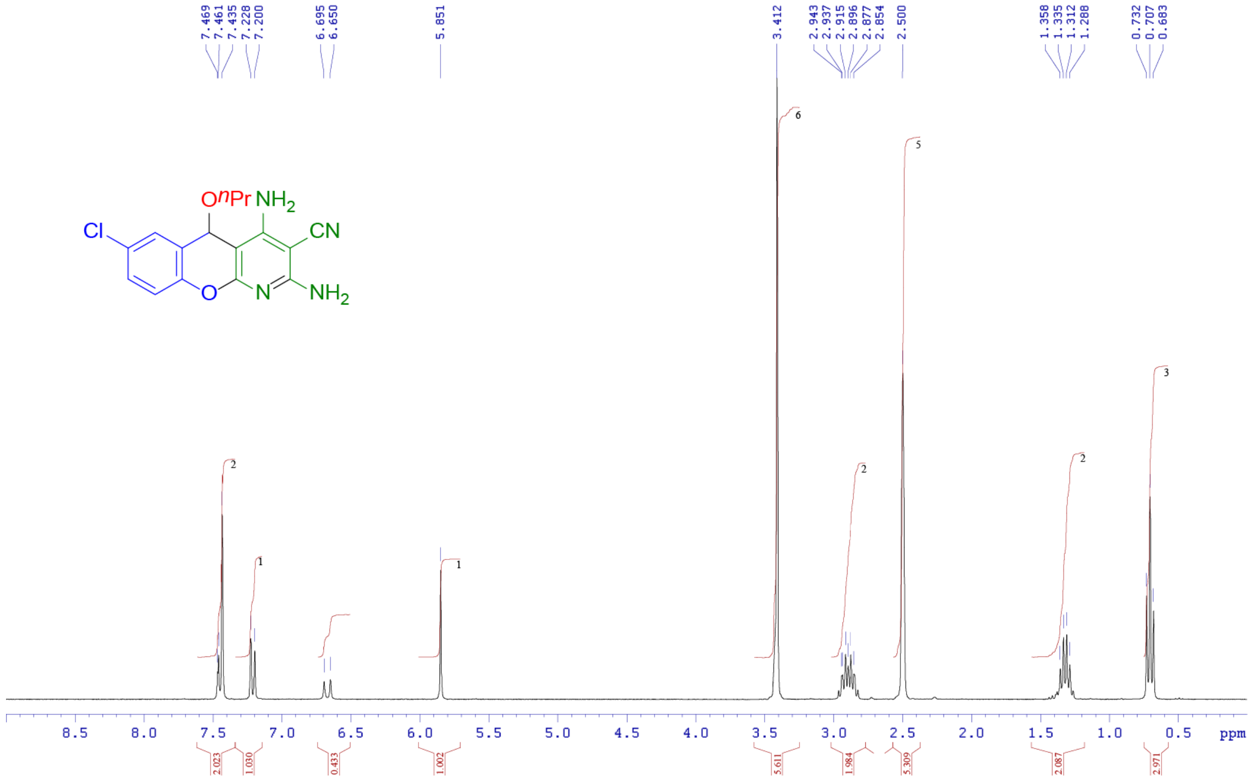

Figure S18. ^{13}C NMR spectrum of 2,4-diamino-7-chloro-5-ethoxy-5*H*-chromeno[2,3-*b*]pyridine-3-carbonitrile **4i** in DMSO-*d*6.

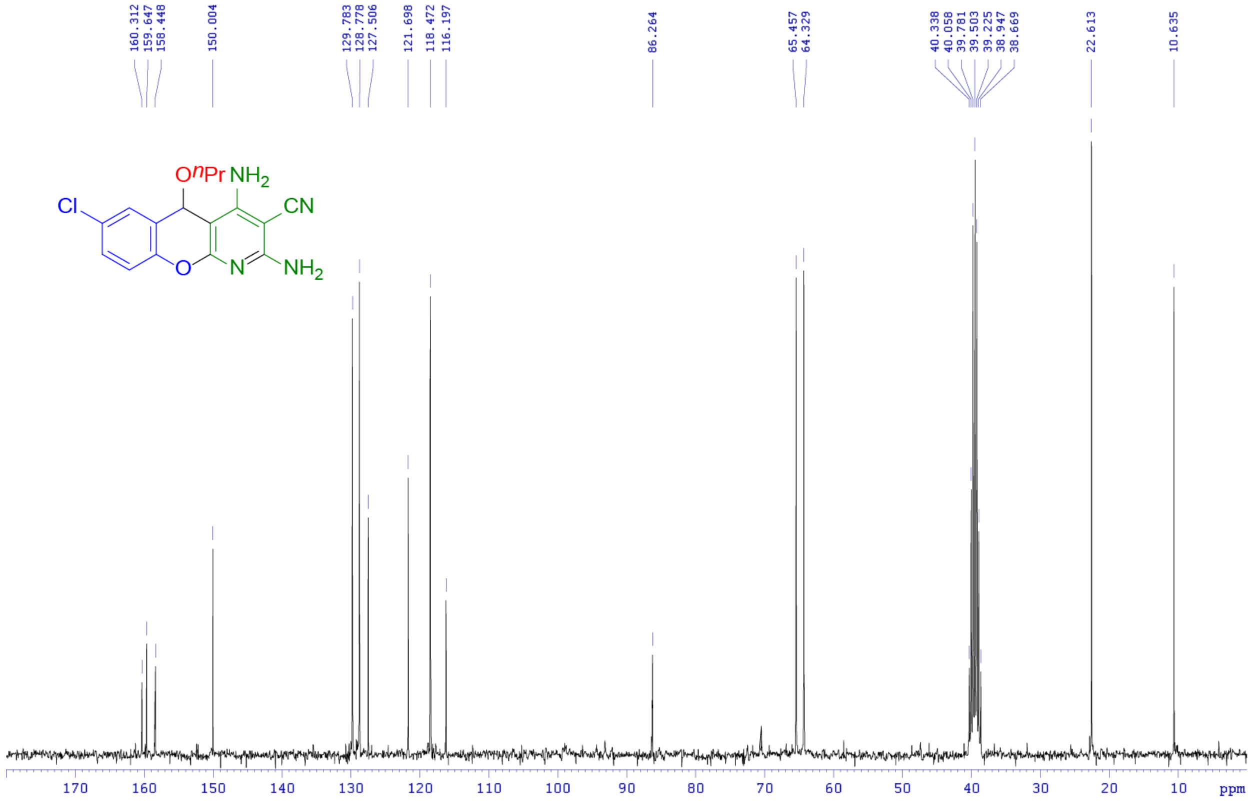

2. Detailed 1D ^{1}H and ^{13}C NMR spectra for compound 4d

Figure S19. Detailed ^{1}H NMR spectrum of 2,4-diamino-5,8-dimethoxy-5*H*-chromeno[2,3-*b*]-pyridine-3-carbonitrile **4d** in DMSO-d_6 (500 MHz, 298 K).

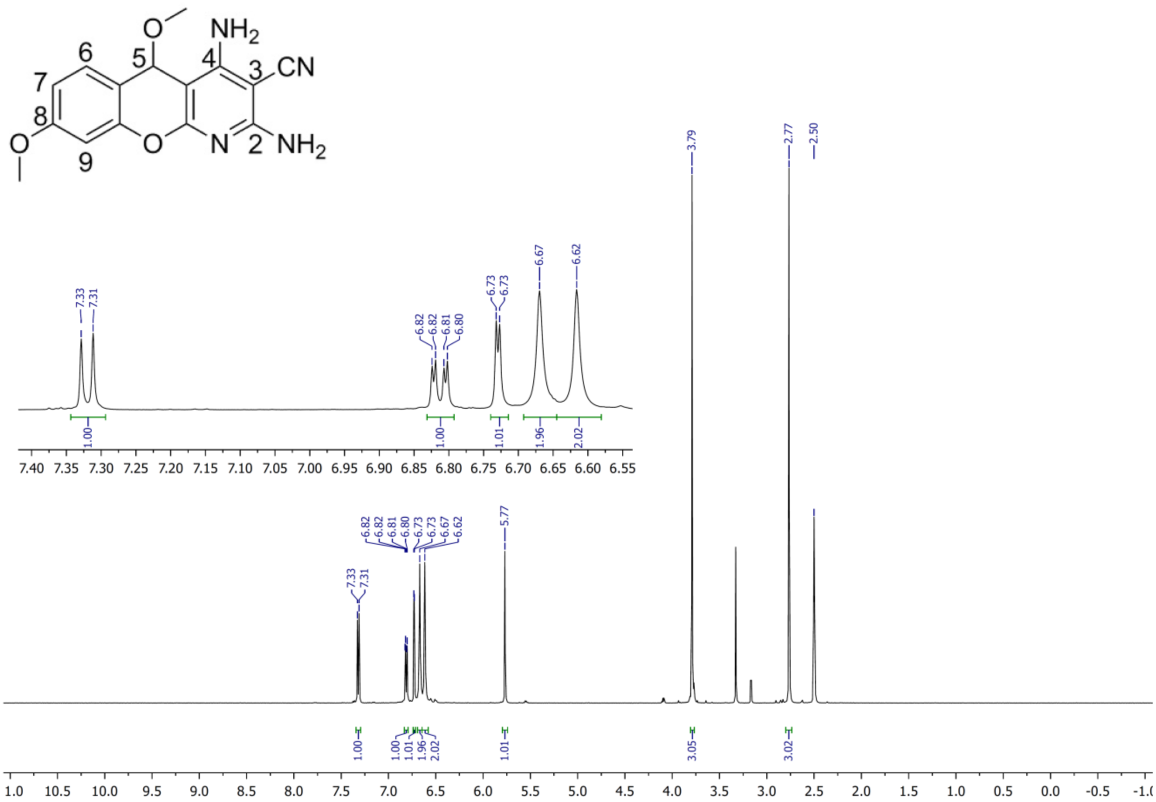

Figure S20. Detailed ^{13}C NMR spectrum of 2,4-diamino-5,8-dimethoxy-5*H*-chromeno[2,3-*b*]-pyridine-3-carbonitrile **4d** in DMSO-d_6 (126 MHz, 298 K).

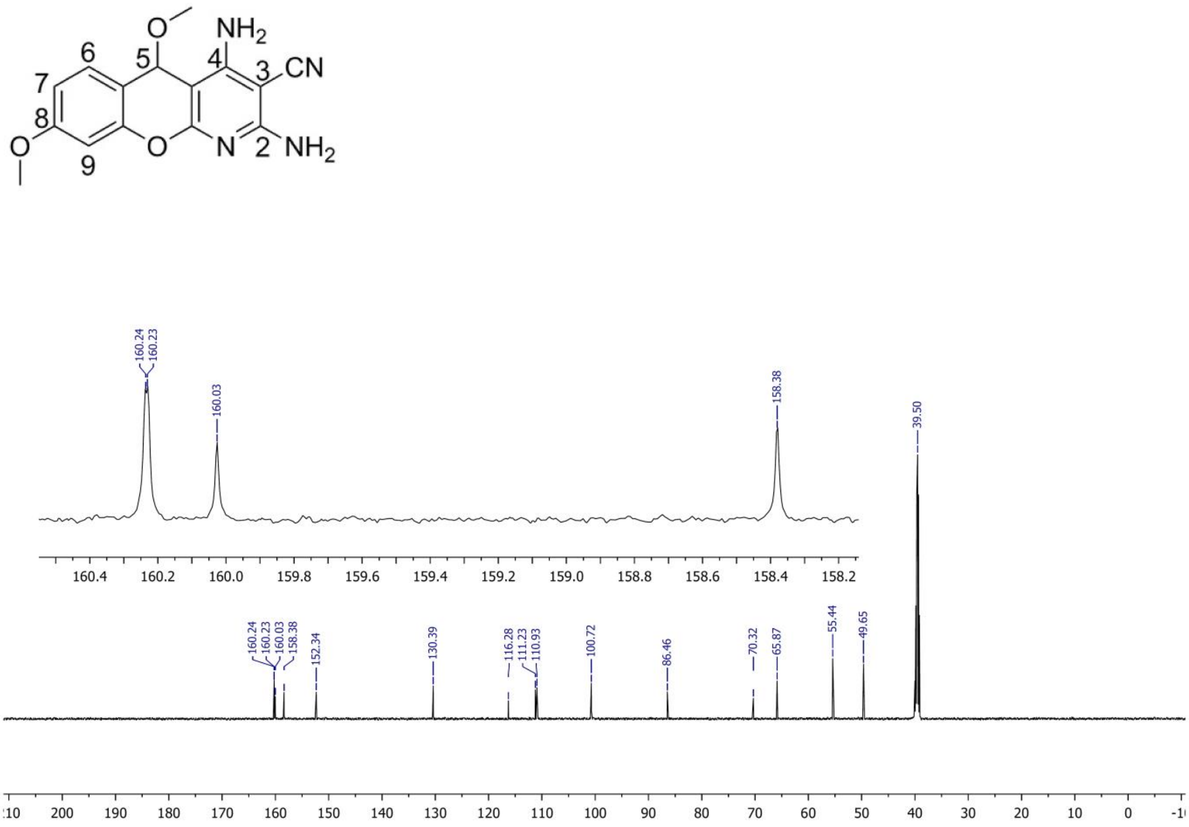

3. 2D NMR data for compound 4d

Figure S21. ^{1}H-^{13}C HMBC NMR spectrum of 2,4-diamino-5,8-dimethoxy-5*H*-chromeno[2,3-*b*]-pyridine-3-carbonitrile **4d** in DMSO-d_6 (400 MHz, 298 K).

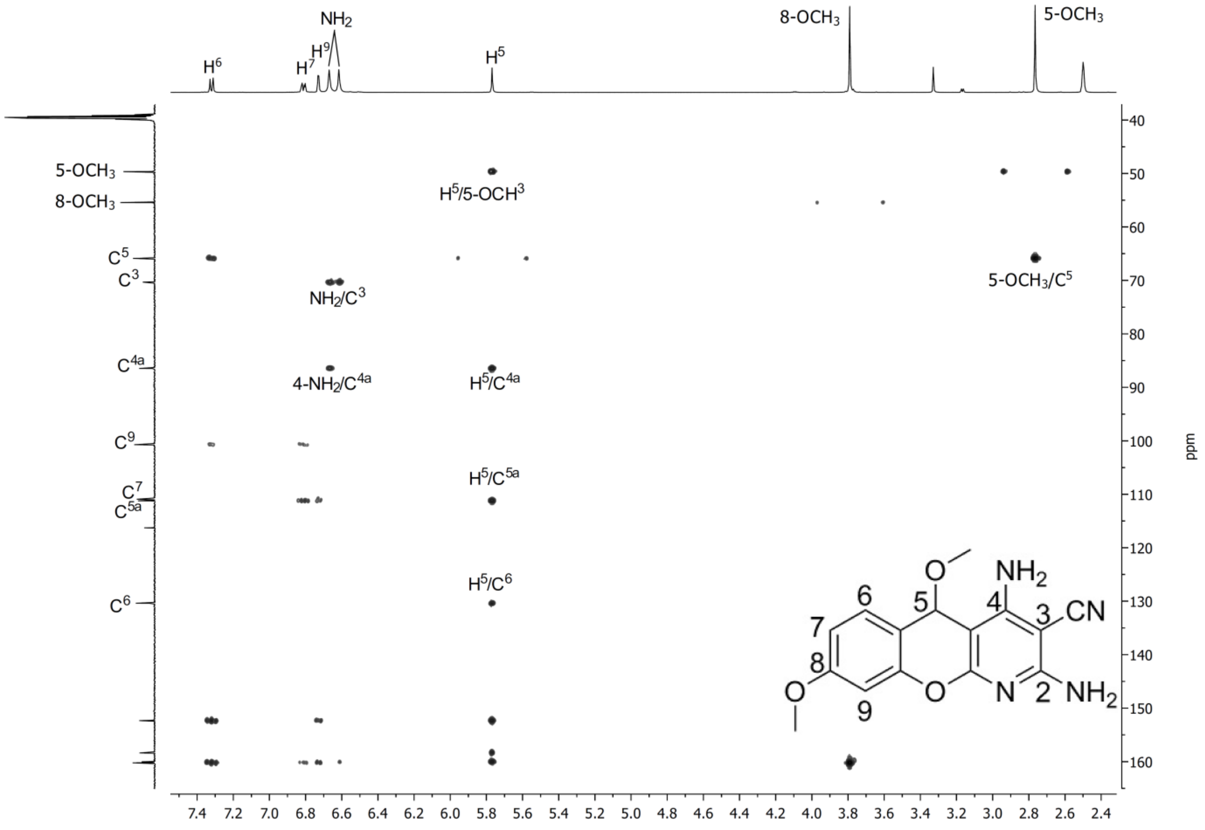

Figure S22. ^{1}H-^{13}C HSQC NMR spectrum of 2,4-diamino-5,8-dimethoxy-5*H*-chromeno[2,3-*b*]pyridine-3-carbonitrile **4d** in DMSO-d_6 (400 MHz, 298 K).

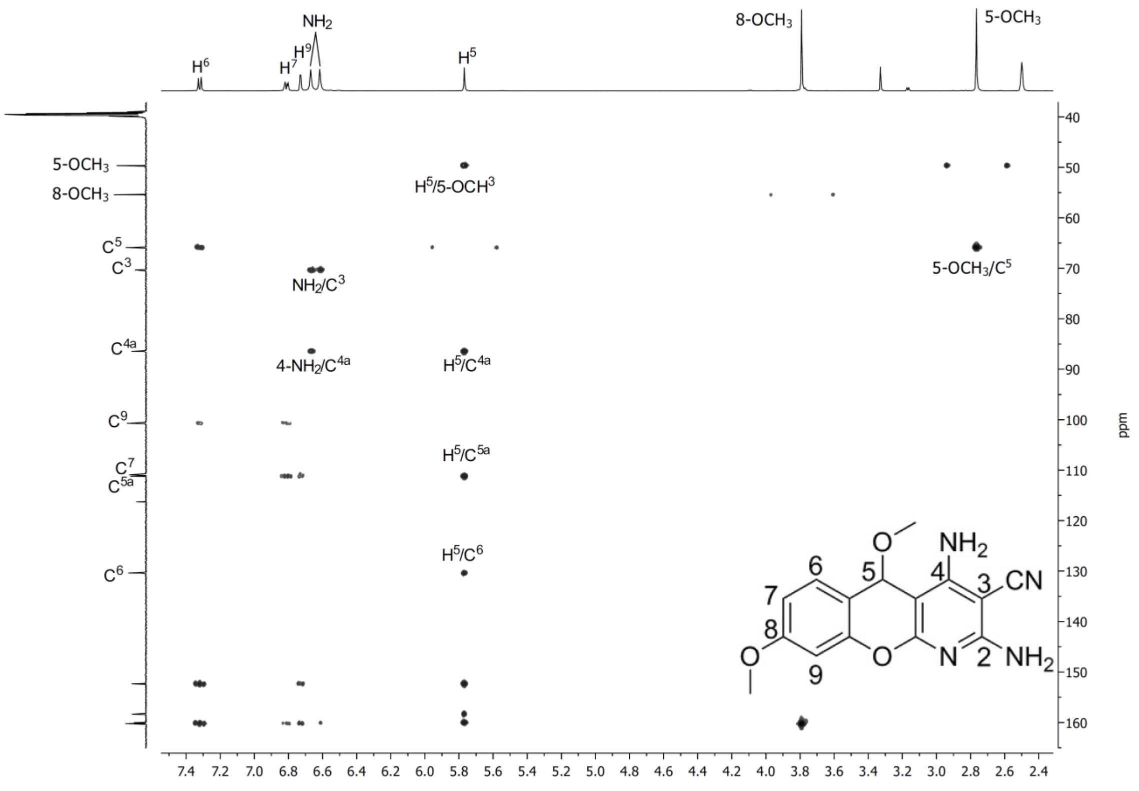

Supplement: Supplementary file 1 [file molecules-28-00064-s001.zip › molecules-2093954-supplementary.pdf]
